# Expression of Tas1 Taste Receptors in Mammalian Spermatozoa: Functional Role of Tas1r1 in Regulating Basal Ca^2+^ and cAMP Concentrations in Spermatozoa

**DOI:** 10.1371/journal.pone.0032354

**Published:** 2012-02-29

**Authors:** Dorke Meyer, Anja Voigt, Patricia Widmayer, Heike Borth, Sandra Huebner, Andreas Breit, Susan Marschall, Martin Hrabé de Angelis, Ulrich Boehm, Wolfgang Meyerhof, Thomas Gudermann, Ingrid Boekhoff

**Affiliations:** 1 Walther-Straub Institute of Pharmacology and Toxicology, Ludwig-Maximilians-University, Munich, Germany; 2 German Institute of Nutrition, Potsdam-Rehbruecke, Germany; 3 Institute for Neural Signal Transduction, Center for Molecular Neurobiology, Hamburg, Germany; 4 Institute of Physiology, University of Hohenheim, Stuttgart, Germany; 5 Institute of Experimental Genetics, Helmholtz-Zentrum, Munich, Germany; Duke University, United States of America

## Abstract

**Background:**

During their transit through the female genital tract, sperm have to recognize and discriminate numerous chemical compounds. However, our current knowledge of the molecular identity of appropriate chemosensory receptor proteins in sperm is still rudimentary. Considering that members of the Tas1r family of taste receptors are able to discriminate between a broad diversity of hydrophilic chemosensory substances, the expression of taste receptors in mammalian spermatozoa was examined.

**Methodology/Principal Findings:**

The present manuscript documents that Tas1r1 and Tas1r3, which form the functional receptor for monosodium glutamate (umami) in taste buds on the tongue, are expressed in murine and human spermatozoa, where their localization is restricted to distinct segments of the flagellum and the acrosomal cap of the sperm head. Employing a Tas1r1-deficient mCherry reporter mouse strain, we found that Tas1r1 gene deletion resulted in spermatogenic abnormalities. In addition, a significant increase in spontaneous acrosomal reaction was observed in Tas1r1 null mutant sperm whereas acrosomal secretion triggered by isolated *zona pellucida* or the Ca^2+^ ionophore A23187 was not different from wild-type spermatozoa. Remarkably, cytosolic Ca^2+^ levels in freshly isolated Tas1r1-deficient sperm were significantly higher compared to wild-type cells. Moreover, a significantly higher basal cAMP concentration was detected in freshly isolated Tas1r1-deficient epididymal spermatozoa, whereas upon inhibition of phosphodiesterase or sperm capacitation, the amount of cAMP was not different between both genotypes.

**Conclusions/Significance:**

Since Ca^2+^ and cAMP control fundamental processes during the sequential process of fertilization, we propose that the identified taste receptors and coupled signaling cascades keep sperm in a chronically quiescent state until they arrive in the vicinity of the egg - either by constitutive receptor activity and/or by tonic receptor activation by gradients of diverse chemical compounds in different compartments of the female reproductive tract.

## Introduction

During their journey through the female genital tract, mammalian sperm are exposed to a wide range of compounds of different origins and chemical properties [1]: From the anterior vagina towards the mature oocyte in the fallopian tube of the oviduct, ejaculated sperm have to sense slight variations in the composition of diverse environmental chemical cues in the different fluids of the female genital tract, like changes in the concentrations of carbohydrates [2], different levels of single amino acids [3], [4], or variations in ion composition and pH [5], [6].

For the essential proper chemical communication with the egg's environment, but also with the oocyte itself, sperm are functionally reprogrammed or capacitated within the female's genital tract [7], [8], [9]. Among other changes, this capacitation-dependent priming enables sperm to perceive gradients of chemo-attractants in the ampullary part of the fallopian tube, secreted by the egg and/or its surrounding structures (chemotaxis) (for review see [10], [11], [12]). In addition to chemosensory capabilities, capacitation endows sperm with the ability to specifically interact with the egg's *zona pellucida* (ZP), a thick extra-cellular glycoprotein matrix surrounding the egg (for review see [13], [14]). However, despite the fundamental importance of detecting diverse chemical ligands for proper sperm function, our current knowledge about the molecular identity of chemosensory receptors on the sperm surface is still rudimentary. This notion holds true for promising ZP-receptor candidates [15], [16], but also for receptor proteins which are able to detect chemical compounds in the different fluids of the female reproductive tract or chemo-attractive cues responsible to successfully guide sperm towards the egg. Although olfactory receptors [17] expressed in the sperm flagellum of different mammalian species [18], [19], [20] paved the way for a new area of research, because they are promising molecular sensors for the various stages of chemical pre-fusion sperm-egg communication steps [21], [22], [23], physiologically relevant ligands for olfactory receptors have not yet been identified (for review see [24]). Moreover, chemical orientation within the female tract entirely occurs in an aqueous environment, whereas olfactory receptors usually detect volatile, lipophilic substances [25], which are unlikely to be dissolved in appropriate concentrations in this aqueous milieu.

Taste receptors of the Tas1 family, which were found to form functional receptors by specific pair-wise dimerization, are specialized to detect hydrophilic ligands as diverse as sugars, artificial sweeteners, sweet proteins (e. g. thaumatin and monellin) and single amino acids, like monosodium glutamate, the savoury taste associated with protein-rich foods (“umami” taste) (for review see [26], [27], [28], [29]). Extra-oral taste receptor expression has been described in several recent reports [30], [31], [32], [33], [34], [35], [36]. Furthermore, the G protein α-subunit gustducin, first identified in taste cells of the tongue [37], was also found to be expressed in mammalian spermatozoa [38], raising the possibility that taste receptors may act as molecular sensors during the sperm's passage through the female reproductive tract. Combining complementary molecular, cellular and reproductive biology approaches we found that the two subunits of the umami taste receptor dimer (Tas1r1/Tas1r3) are expressed in mouse and human spermatozoa. Furthermore, using a Tas1r1/mCherry reporter mouse line we observed that Tas1r1 null mutant sperm display a higher rate of spontaneous acrosome reaction and an elevated level of [Ca^2+^]_i_ (intracellular calcium concentration)and cAMP (cyclic adenosine monophosphate). Because taste receptors may be permanently activated by compounds in the surrounding environment of the female reproductive tract, we hypothesize that these chemosensory receptors constantly suppress Ca^2+^ and cAMP-triggered maturation processes during the sperm's journey towards the egg.

## Results

### Transcripts of Tas1r Taste Receptors in Murine Testis

**Figure 1:**
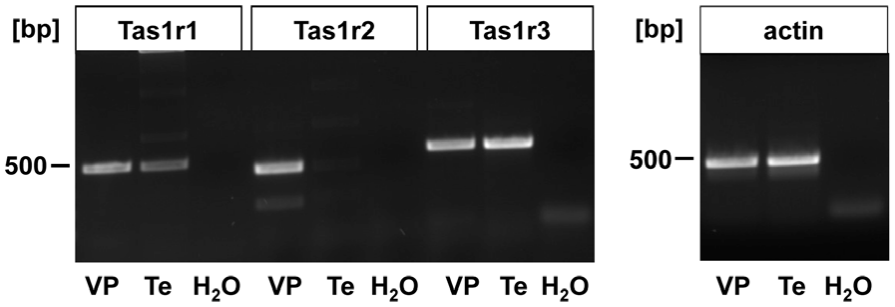
Detection of Tas1r-transcripts from cDNA of murine vallate papillae and testicular tissue using RT-PCR. Primer sets specific for the murine Tas1r1 and Tas1r3 yielded amplification products with the expected size ([*Tas1r1*]; 468 bp; ([*Tas1r3*]; 510 bp) from cDNA derived from taste [VP] as well as from testicular tissue ([*Te*]), whereas the primer pair for the Tas1r2 only resulted in the generation of an amplification product in taste cDNA ([*Tas1r*2]; 403 bp [*VP*]), but not in testicular cDNA ([*Te*]). cDNA quality was assured determining amplification products with a primer pair against the housekeeping gene beta-actin (right panel, [*actin*]; 425 bp]). Negative controls present samples in which water was used instead of cDNA ([*H_2_O*]). The identities of amplified taste receptor subtypes are indicated on the top of each panel. The corresponding 500 bp DNA size marker is shown on the left of both panels.

To determine whether members of the Tas1 taste receptor family are expressed in mammalian germ cells, we subjected reverse-transcribed murine testicular mRNA to PCR analysis using specific primer pairs based on published mouse Tas1 receptor sequences. We started with control experiments verifying that isolated mRNA was not contaminated with genomic DNA. PCR-reactions with L8 primers (data not shown) and those with a β-actin primer pair set (Fig. 1; right panel, [*actin*]) resulted in amplification fragments of the predicted size without any additional amplification products, thus ensuring that genomic cDNA would not lead to erroneously positive RT-PCR-results. Quality-controlled cDNA from testicular tissue ([*Te*]) and taste bud-derived cDNA (from vallate papillae, [*VP*]), applied as positive control, were then used to examine whether transcripts of the Tas1r family of taste receptors were present in reproductive tissue. The results shown in figure 1 (left panel) document that application of specific primer pairs for the umami taste receptor Tas1r1 ([*Tas1r1*]) and the pivotal dimerization partner for the sweet and the umami taste receptor Tas1r3 ([*Tas1r3*]) yielded amplification signals of the expected size in cDNA from mouse taste papillae and from testis-derived cDNA. Subsequent sub-cloning and sequencing of the obtained PCR fragments confirmed the sequence identity with previously published murine Tas1r1 (GenBank accession no AY032623) and Tas1r3 sequences (accession no NM0311872). However, in contrast to recently published data [39], we were not able to amplify transcripts of the sweet taste receptor Tas1r2 (accession no. AY0326229) from mouse testicular cDNA (Fig. 1; [*Tas1r2*], [*Te*]), although three independent primer pairs were employed, each successfully working on cDNA derived from taste tissue (for representative s. Fig. 1; left panel; [*Tas1r2*], [*VP*] and Fig. S1). Thus, Tas1r2 mRNA levels appear to be very low in testicular tissue.

### Expression of Tas1r1 and Tas1r3 Receptor Proteins in Mammalian Spermatozoa

**Figure 2:**
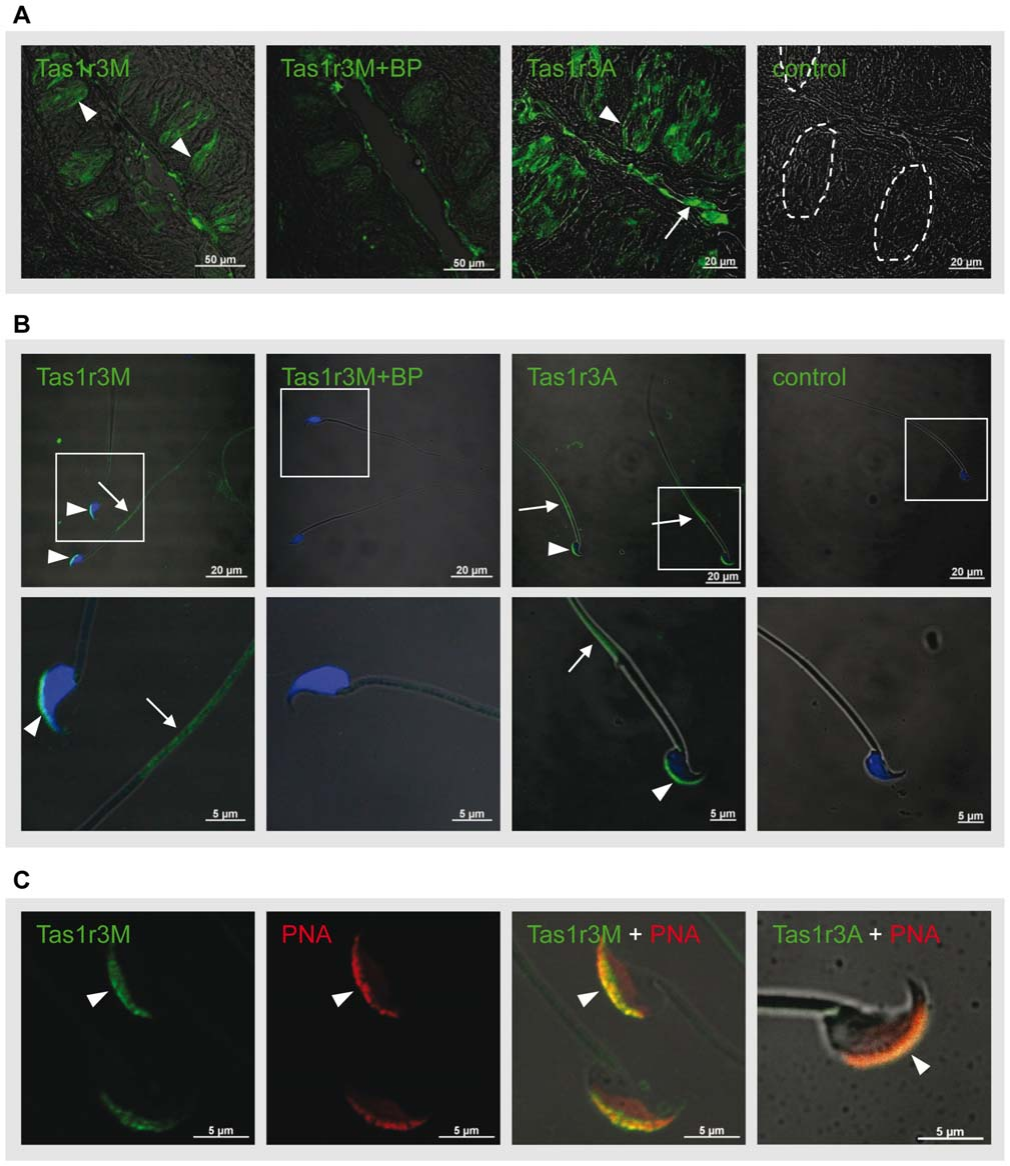
Expression of Tas1r3 in murine taste buds and epididymal spermatozoa. [**A**] Immunohistochemical analysis of Tas1r3 localization in taste cells of murine vallate papillae. The two applied Tas1r3-specific antisera ([*Tas1r3M*]; [*Tas1r3A*]) labeled a subset of spindle-shaped cells within taste buds (arrowheads); neutralization of the Tas1r3M primary antiserum with an excess of the corresponding antigenic peptide ([*Tas1r3M+BP*]) resulted in elimination of the fluorescence signals. Sections incubated with the secondary antiserum alone showed no immunoreactivity (left panel; [*control*]). The dotted lines in the control panel highlight the border of individual taste buds. [**B**] Subcellular localization of Tas1r3 in murine spermatozoa determined by indirect immunofluorescence. Isolated murine sperm were fixed with ice-cold methanol and subsequently incubated with one of the two above mentioned Tas1r3 antisera ([*Tas1r3M*]; [*Tas1r3A*]). Bound primary antiserum was visualized by a FITC-conjugated anti-rabbit IgG. Nuclear staining was performed with propidium iodide (shown in blue). An application of both Tas1r3 antisera resulted in a strong immunostaining (green fluorescence) which was restricted to the convex side of the sperm head (arrowheads) and the principle piece of the sperm flagellum([*Tas1r3M*] and [*Tas1r3A*], arrows). Pre-incubation of the Tas1r3M antiserum with the immunogenic peptide completely prevented the immunoreactivity ([*Tas1r3M+BP*]). Negative controls represent samples incubated with the secondary antiserum alone (right panel; [*control*]). The inserts in the upper panels show regions presented at higher magnifications in the micrographs below. [**C**] Acrosomal localization of Tas1r3 in murine sperm. To determine the precise subcellular localization of Tas1r3 in mouse spermatozoa, freshly isolated epididymal mouse sperm were probed with one of the two rabbit anti-Tas1r3 antisera ([*Tas1r3M*], [*Tas1r3A*]) (green) and the acrosomal marker peanut agglutinin ([*PNA*]) conjugated to TRITC (red). Note that overlay of each of the two antiserum-derived fluorescence staining patterns with the labeling signals of the fluorochrome-conjugated PNA resulted in an orange-yellow fluorescence color in the acrosomal cap ([*Tas1r3M+PNA*]; [*Tas1r3A+PNA*] arrowhead), indicating a localization of the Tas1r3 within the acrosomal region. Presented experiments show representative results of experiments which were repeated at least three times with different tissue and cell preparation.

So far, our results indicate the presence of Tas1r1 and Tas1r3 transcripts in testicular tissue, whereas Tas1r2 was not detectable. To test whether the identified Tas1r family members are actually translated in male germ cells, antisera specific for rodent taste receptors were required. First, we evaluated the specificity of available antisera by determining their immunostaining patterns on cryostat sections of mouse and rat vallate and fungiform papillae. Two commercially available anti-Tas1r1 and anti-Tas1r2 antisera (Santa Cruz) recommended to detect rodent taste receptor subtypes, did not yield specific immunolabeling on mouse and rat taste tissue in our hands (data not shown). Therefore, our approach was restricted to the use of two Tas1r3 antisera, whose staining pattern was found to be essentially identical to the described labeling of Tas1r3 probes on sensory cells of vallate papillae: An anti-Tas1r3 specific antiserum generated against amino acids 239–255 of the murine Tas1r3 receptor protein, named anti-Tas1r3M [40], and a commercially available Tas1r3 specific antiserum, termed anti-Tas1r3A in this manuscript (Abcam). Figure 2A documents the results of control experiments using sections of vallate papillae of the murine tongue. Incubation of sections of taste tissue with the Tas1r3M antiserum (left panels; [*Tas1r3M*], arrowhead) resulted in intense immunostaining of spindle-shaped cells within taste buds as described previously [53], [141]. There was a partial overlap with the expression pattern of α-gustducin, routinely used as a positive control in immunohistochemical experiments (data not shown). Furthermore, when testing for specificity, the primary antiserum was neutralized by an excess of the immunogenic peptide and the anti-Tas1r3M IgG-derived immuno-signals were completely abolished (Fig. 2A; [*Tas1r3M+BP*]). Employing the second Tas1r3 antiserum, a comparable staining pattern was detected: Incubation with the Tas1r3A antiserum yielded immuno-positive signals which were concentrated to a subset of elongated cells within the taste bud (Fig. 2A; [*Tas1r3A*], arrowhead), apart from some faint unspecific staining by the antiserum in the cleft of the papilla (Fig. 2A; [*Tas1r3A*], arrow).

To examine Tas1r3 receptor protein expression in mature mouse germ cells, we immunostained isolated epididymal sperm using the suitable anti-Tas1r3 antisera. To accentuate the typical sub-cellular compartmentalization of the sperm, nuclei were counterstained with the DNA-intercalating dye propidium iodide. Figure 2B shows that epididymal mouse sperm exposed to the Tas1r3M antiserum exhibited an Fluorescein isothiocyanate (FITC)-derived fluorescent pattern in both cellular compartments of the sperm (Fig. 2B; [*Tas1r3M*]) which was abolished by its immunogenic peptide (Fig. 2B; [*Tas1r3M+BP*]). Labeling of the sperm tail was limited to the principal piece of the flagellum (Fig. 2B; [*Tas1r3M*], arrow), where α-gustducin is localized as well [38]. In addition, a bright green staining was detected in the hook-shaped acrosomal structure of the sperm head, not overlapping with the propidium iodide fluorescence (Fig. 2B; [*Tas1r3M*]; arrowheads). A similar labeling pattern was obtained when the other Tas1r3 specific IgG was applied (Fig. 2B; [*Tas1r3A*]): Tas1r3A-IgG resulted in immunostaining of the head only visible in the acrosomal crescent (Fig. 2B; [*Tas1r3A*]; arrowhead). The immunoreactivity in the sperm flagellum was restricted to the principal piece (Fig. 2B; [*Tas1r3A*]; arrows) whereas other tail segments such as the mid- and endpiece region did not show any labeling. To confirm the observed acrosomal localization of the identified taste receptor proteins, we performed double labeling experiments using the acrosomal lectin marker peanut agglutinin (PNA) [41]. Overlay of labeling signals obtained for both Tas1r3 specific antisera and a fluorochrome (TRITC)-conjugated PNA led to a coincident yellow crescent-shaped staining pattern (Fig. 2C; [*Tas1r3M+PNA*] and [*Tas1r3A+PNA*]; arrowheads), thus confirming the localization of the Tas1r3 immunoreactivity to the acrosomal cap of mouse spermatozoa.

### Analysis of Tas1r1 and Tas1r3 Expression in Mouse Testis using a novel knock-in Tas1r1-mCherry Reporter Mouse Strain

**Figure 3:**
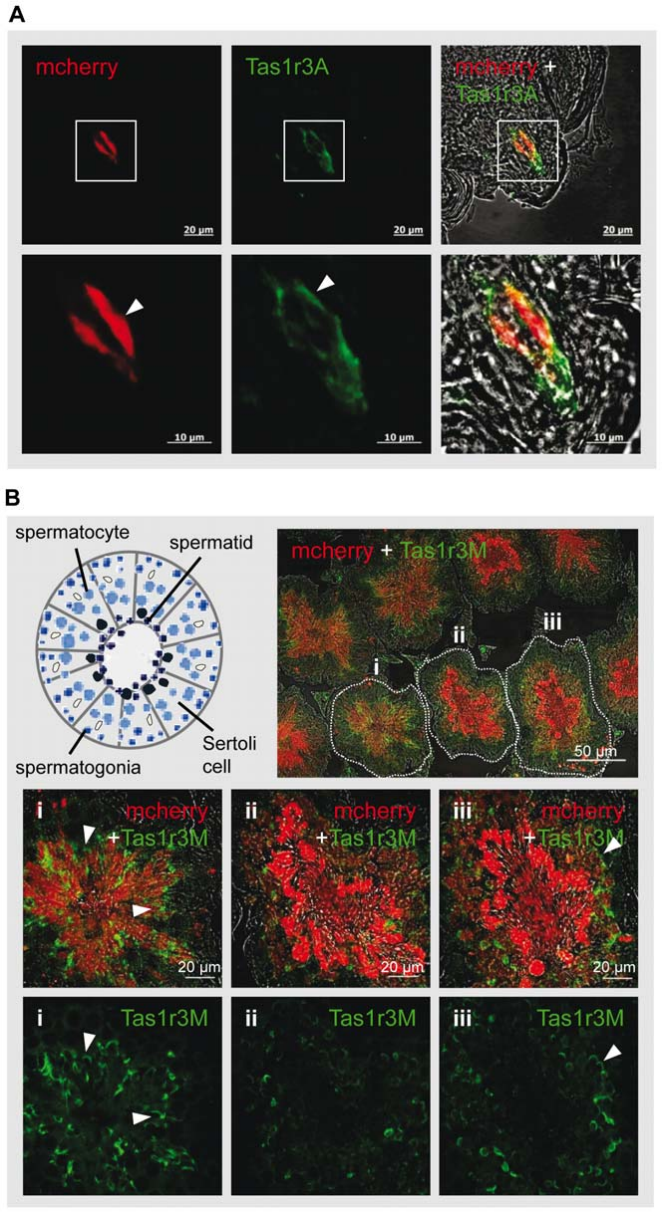
Tas1r1 mCherry reporter expression and co-localization with Tas1r3. [**A**] Localization of the Tas1r1 reporter protein mCherry and the Tas1r3 receptor in a fungiform papilla of the tongue. Coronal sections of a fungiform papilla of a Tas1r1/mCherry reporter mouse were incubated with a Tas1r3 specific antiserum ([*Tas1r3A*]) which was visualized using a FITC-coupled secondary antiserum (green). Subsequently, fluorescence labeling patterns were imaged using confocal microscopy. Note that mCherry fluorescence (red), reflecting activity of the Tas1r1 promoter in the taste bud, and staining with the Tas1r3 specific antiserum are visible in the same cells of the papilla (right panels; ([*mCherry+Tas1r3A*]). However, while the mCherry fluorescence signal is located in the cytoplasm of the immune-positive cells (lower left panel; [*mCherry*], arrowhead), the Tas1r3 immunostaining is mainly observed at the plasma membrane (lower middle panel; [*Tas1r3A*], arrowhead). The superimposed boxes in upper panels represent higher magnifications shown in lower panels. [**B**] Tas1r1mCherry reporter expression and co-localization with Tas1r3 in testicular tissue. In the upper left panel, a schematic drawing of a single seminiferous tubule with different stages of developing germ cells during spermatogenesis is shown. Note that germ cells of a distinct developmental stage are organized in concentric layers within the tubule: In the most basal cell layer, the spermatogonial stem cells (middle blue) are located, followed by spermatocytes (light blue), round spermatids and finally the most mature elongating spermatids concentrated in the luminal region of the tubule (dark blue). Monitoring localization of the taste dimerization partner by applying a Tas1r3 specific IgG ([*Tas1r3M*]; green). mCherry expressing tubules also showed immunoreactivity for the Tas1r3 antiserum ([*mCherry+Tas1r3M*]). The dotted lines in the overview in the top panel mark higher magnifications of three representative tubules with distinct combination of germ cell generations depicted below ([*i*], [*ii*], [*iii*]). Pictures of the fluorescence channels (green, [*Tas1r3M*]; red, [*mCherry*]) are merged with the corresponding transmitted-light channels, in the lower panels, only the FITC-derived fluorescence is shown. Micrographs show representative pictures of different Tas1r1/mCherry male mice with comparable results.

To investigate Tas1r1 receptor expression in male germ cells and to elucidate the putative role of Tas1r in reproduction, we took advantage of a Tas1r1-mCherry reporter mouse line carrying a recombinant *Tas1r1* allele, in which the Tas1r1 open reading frame was replaced by a red monomeric cherry fluorescent protein (mCherry) expression cassette. This reporter mouse strain allows to examine the effect of receptor deficiency on reproduction, and in addition permits to detect Tas1r1 expression in extra-oral tissues, such as reproductive organs. We initially examined whether the mCherry reporter protein is detectable in the same taste bud cells as the endogenous Tas1r1 receptor [42], [43], [44] and found mCherry to be present in single spindle-shaped cells of fungiform papillae (Fig. 3A, [*mCherry*]), thus confirming cell-type-specific expression of the reporter gene which is comparable to the endogenous taste receptor protein expression pattern. Since in taste buds, Tas1r1 dimerizes with the Tas1r3 protein to form a functional umami receptor (for review see [27], [45], [46]), we determined the distribution of the Tas1r3 receptor protein in single taste buds in combined immunohistochemical approaches. Using coronal sections of taste tissue of Tas1r1 mCherry reporter mouse line and a Tas1r3 specific antiserum (Fig. 2A; [*Tas1r3A*]), green Tas1r3-derived immunofluorescence was detected in the same cells as the mCherry fluorescence (Fig. 3A, [*mCherry+Tas1r3A*]). This observation supports the notion that the created knock-in mouse line is suitable to examine extraoral [47] *in vivo* expression of the Tas1r1 receptor. However, the fluorescence staining pattern of the two taste receptor markers showed a different sub-cellular distribution in the stained sensory cells: Whereas Tas1r3 immunoreactivity was mainly concentrated at the cell membrane of the taste cells (Fig. 3A; middle panel; [*Tas1r3A*]; arrowhead), mCherry fluorescence was primarily localized to the cytoplasm (Fig. 3A; left panel; [*mCherry*]; arrowhead).

**Figure 4:**
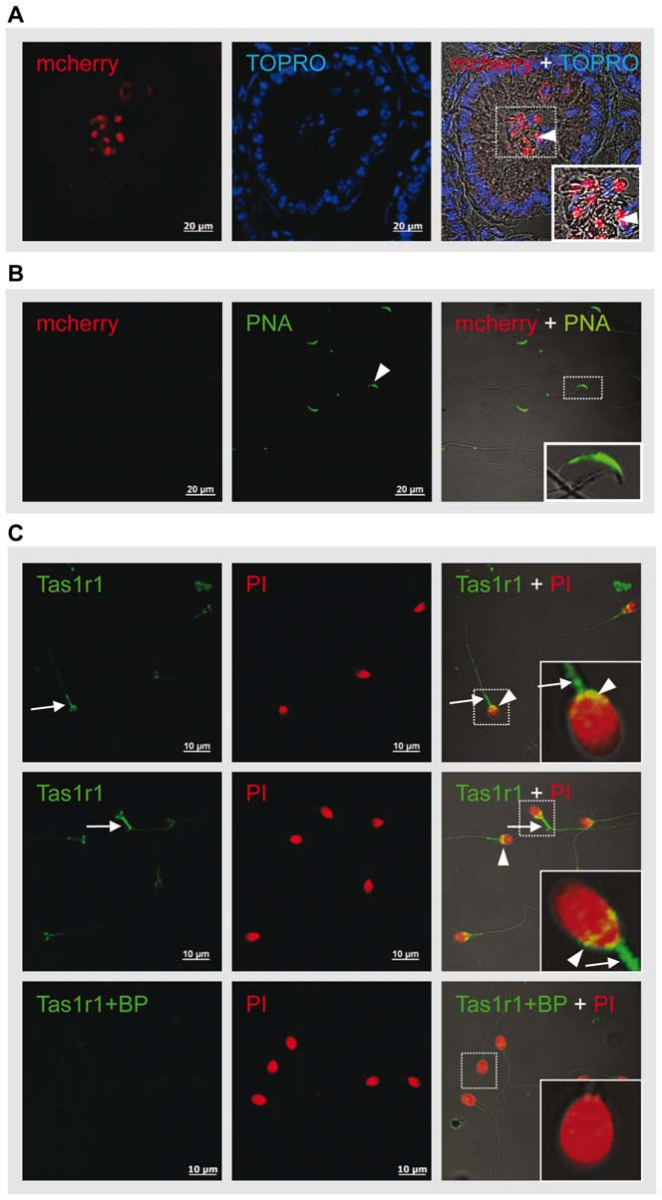
Tas1r1 expression in mammalian spermatozoa. [**A**] Extrusion of the mCherry protein during sperm maturation in the epididymis. Cryosections of the caput of the epididymis of a Tas1r1/mCherry reporter mouse were incubated with an anti-mCherry antiserum (red; [*mCherry*]) and counterstained with the nuclear dye TO-PRO-3 (blue; [*TOPRO*]). ([*mCherry+TOPRO*], inset, arrowhead). [**B**] mCherry fluorescence is not detectable in mature epididymal sperm. Isolated sperm of the mutant mouse line were fixed with PFA and counterstained with the FITC-coupled acrosomal marker PNA (middle panel; arrow; [*PNA*]). Imaging sperm for mCherry fluorescence revealed that the fluorescent protein was completely lost during epididymal maturation (left panel [*mCherry*]). Insets in the right panels show higher magnification of the tubule's lumen [**A**] or a sperm's acrosome [**B**], respectively. [**C**] Expression of Tas1r1 in human spermatozoa. Ejaculated human sperm were incubated with a human specific Tas1r1 antiserum; bound primary antiserum was visualized applying a FITC-conjugated anti-rabbit IgG. The two representative confocal micrographs document that the anti-Tas1r1 IgG ([*Tas1r1*]) showed a staining in the flagellum (arrow) and in the post-acrosomal region as well as at the equatorial segment (arrowheads). Immunostaining in both subcellular compartments was extinguished upon neutralizing the primary antiserum with an excess of the corresponding immunogenic peptide (lower panels; [*Tas1r1+BP*]), thus confirming specificity of the detected immunolabeling. Negative controls, in which the primary antiserum was omitted, did not show any labeling (data not shown). Confocal images were produced by an overlay of corresponding fluorescence channels (propidium iodide, [red]; FITC-conjugated secondary antiserum, [green]) and the transmission channel. Boxes indicate regions that are magnified in insets in the right panels. Experiments were repeated with at least three independent sperm preparations from different donors, showing comparable results.

To confirm our immunocytochemical results of Tas1 receptor expression in mature spermatozoa, we monitored the expression of mCherry in reproductive tissues. Therefore, testis sections were prepared from Tas1r1 mCherry knock-in mice and imaged for color-coded cells (Fig. 2B). Spermatogenesisis is characterized by a series of mitotic divisions with distinct stages of differentiating germ cells localized to defined concentric bands of the seminiferous tubules (s. Fig. 3B; schematic drawing in the left panel in the top): Spermatogonia are located in the basal cell layer, followed by two meiotic spermatocyte division stages and finally haploid spermatids accumulating in the central cell layer of the tubular unit (for review see [48]). Due to this defined spatial organization, mCherry fluorescence signals in testicular tissue sections allow to determine at which developmental stages the receptor is expressed. Moreover, performing combined immunostaining approaches, it is feasible to simultaneously investigate the spatial expression profile of the tongue-specific dimerization partner of Tas1r1, Tas1r3, in spermatozoa (s. Fig. 2A and B). mCherry fluorescence signals were found in all analyzed seminiferous tubules of cross sections of testicular tissue of the Tas1r1 reporter mouse strain (s. overview in Fig. 3B; top panel on the right). Comparing mCherry appearance in single tubules, which typically display one of twelve characteristic combinations of distinct phases of differentiating germ cells [49], fluorescence signals were always detected in more mature round and elongated spermatids in the tubular lumen, whereas sparse fluorescence was detected in the periphery, where the early stages of spermatogenesis occur (Fig. 3B). Tas1r3 immunoreactivity was visible in all tubules examined (Fig. 3B; middle and bottom panels, [*i*], [*ii*], [*iii*]). Moreover, we observed that the Tas1r3 receptor emerges at the same phases of spermatogenesis as the Tas1r1 reporter protein: Tas1r3-derived FITC-labeling was most prominent in cells of the luminal layers of the tubular units, where late spermatocytes and spermatids are concentrated, while no obvious staining was observed in spermatogonia and early spermatocytes located in the outer tubule regions (Fig. 3B; upper right panel; [*mCherry+Tas1r3M*]). At higher magnification, one can observe that the sub-cellular fluorescence of Tas1r3 did not overlap exactly with the fluorescence pattern of mCherry: While the mCherry signal cannot be attributed to a distinct sub-cellular compartment of developing germ cells, Tas1r3 staining was mainly concentrated in the developing acrosomal region of spermatids (Fig. 3B; middle and bottom panels; [i], [iii]; arrowhead). Since mCherry fluorescence mirrors Tas1r1 promoter activity, the pattern of mCherry labeling might differ from the endogenously expressed receptor protein. However, one may speculate that the artificial and dispensable mCherry protein gets lost in fully developed germ cells. Recent studies showed that final steps of spermatogenesis are accompanied by an extensive extrusion of superfluous cytoplasmic components, which are deposited in detached membrane-limited organelles, subdivided into small compartments designated as residual bodies [50] or larger cytoplasmic droplets [51]. To assess mCherry labeling in late stages, its expression was determined in isolated epididymal sperm of Tas1r1/mCherry knock-in animals (Fig. 4B) and in the epididymis (Fig. 4A), the storage organ of mature spermatozoa [51], respectively. Utilizing the DNA-staining dye TO-PRO-3 ([*TOPRO*]), we detected nucleus-derived fluorescent signals in cells lining the epididymal epithelium and in the lumen of the tubules where mature sperm are located (Fig. 4A; [*TOPRO*]). Thus, Tas1r1 null mutant mice show no obvious morphological defects in the epididymis. However, the luminal mCherry immunoreactivity appears to be accumulated in large vesicular structures most likely representing cellular extrusion organelles (Fig. 4A; [mCherry+TOPRO], arrowhead). Extrusion of the cytoplasmatic mCherry protein was confirmed by monitoring mCherry fluorescence in isolated sperm cells: Whereas the lectin PNA (green fluorescence) labeled a typical crescent-shaped acrosome in Tas1r1 null mutant sperm (Fig. 4A; arrowhead; [*PNA*]), red coloration reflecting the presence of mCherry was not found, even after increasing the sensitivity for mCherry detection by applying an anti-DsRed antiserum (data not shown). Thus, the mCherry fluorescence protein mostly likely represents cellular detritus for germ cells and might be excluded from maturing spermatozoa. However, since it is widely accepted that sperm are transcriptionally and translationally silent [52], proteins essential for a successful fertilization already have to be synthesized during sperm cell development. Therefore, the marked increase in mCherry fluorescence intensity at late stages of spermatogenesis (Fig. 3B) together with the co-localization of its obligatory dimerization partner, the Tas1r3 protein, in mature spermatozoa (Fig. 2B), can reliably be interpreted to indicate the presence of the Tas1r1 receptor protein in fully developed germ cells. However, due to the shortcomings of commercially available antibodies, we were unable to confirm the expression of the Tas1r1 protein in mature sperm, at least in mouse. Due to the availability of reliable functioning antisera against the human Tas1r1 receptor protein, we decided to clarify this point in human sperm cells. To validate the specificity of Tas1r1 antisera of which four had been reported to detect the human umami taste receptor, we transfected HEK293 cells with a human Tas1r1 cDNA fused to a Herpes Simplex Virus (HSV)-tag. In Western blot experiments we found that one tested anti-Tas1r1 IgG (Tas1r1 A, Acris) detected a single immuno-reactive band with the expected size of the Tas1r1 (93 kDa) (Fig. S2A; [*ab*]). This band was also labeled with an anti-HSV antiserum (data not shown) and was eliminated by preincubation with the immunogenic peptide (Fig. S2A, [*ab+bp*]). This Tas1r1 antiserum was subsequently used to analyze umami taste receptor expression in freshly ejaculated human sperm. The antiserum caused immunostaining of human sperm (for representative results s. Fig. 4C; [*Tas1r1*]) that was abolished after neutralizing with the antigenic peptide (Fig. 4C; bottom panel [*Tas1r1+BP*]). At higher magnification, staining was detected in both subcellular compartments of this germ cell type: Labeling of the sperm flagellum was most prominent in the mitochondria-rich mid-piece segment (upper panel in Fig. 4C; arrows) whereas the flagellum's principal and end tail segments only showed faint immunoreactivity. In addition, the post-acrosomal region and the equatorial segment of the paddle-shaped head were labeled (Fig. 4B; higher magnifications in the right panels; arrowhead). Of note, immunostaining of the potential dimerization partner of Tas1r1 in human sperm, using an antiserum which also specifically labeled the recombinant protein in HEK cells (Fig. S2B) [53], revealed a comparable, but slightly broader subcellular expression pattern which also encompassed the acrosomal cap and the sperm flagellum (Fig. S2C). These observations indicate that the two subunits forming the tongue umami taste receptor show an overlapping subcellular distribution pattern in sperm of different mammalian species.

### Reproductive Success and Morphometric Analyses of Reproductive Organs of Tas1r1-deficient Mice

To examine whether taste receptors might play a role in reproduction, we performed breeding experiments using 8–16 week old wild-type ([*+/+*]), Tas1r1 heterozygous ([*+/−*)]), and Tas1r1 homozygous ([*−/−*]) mice. Subsequently, crosses were analyzed for alterations in their reproductive phenotype (Tables 1 and 2). Mice homozygous for the targeted mutation were viable, fertile and normal in overall anatomy and general behavior. Moreover, breeding pairs of Tas1r1-deficient mice were successful in siring litters, with no differences in the survival rate or ratio of male and female offspring (data not shown). Quantifying standard reproductive parameters, knock-out breeding pairs did not display significant differences in pub numbers or in time to delivery pubs (Table 1). Analogous results were obtained comparing the genotype distribution of offspring from heterozygous Tas1r1 mating pairs: No shift in the expected Mendelian 1∶2∶1 ratio of produced offspring was detected (Table 2).

**Table 1:** Reproductive success of homozygote and heterozygote Tas1r1-deficient mice compared to wild-type mice.

	genotype of mating partners		
reproduction parameter	[+/+]×[+/+]	[+/−]×[+/−]	[−/−]×[−/−]
time to litter [d]	29.4±1.7	26.8±1.5	29.5±2.0
time to first litter [d]	26.1±2.3	24.9±1.9	34.6±6.3
litter size [no of pubs]	5.7±0.5	7.0±0.4	6.0±0.3

In a continuous mating study, intervals between mating and delivery of pubs [*time to litter*], time to first delivery [*time to first litter*] and number of weaned pubs per litter [litter size] were determined for wild-type C57BL/6 animals [*(+/+)×(+/+)*] and for Tas1r1 mCherry heterozygous [*(+/−)×(+/−)*] and homozygous [*(−/−)×(−/−)*] breeding pairs. Given data are mean values ± SEM; 7–14 breeding pairs with 31–50 litters were analyzed per genotype; p-values were determined using an unpaired Student's t test (two-tailed).

**Table 2:** Genotype distribution of offspring from heterozygous Tas1r1 mating pairs.

[+/−]×[+/−] mating	number of pubs		
offspring genotype	observed (% of total)	expected (% of total)	X^2^ test
[+/+]	112 (26%)	107 (25%)	
[+/−]	210 (49%)	213 (50%)	P>0.84
[−/−]	105 (25%)	107 (25%)	

Breeding was carried out on a heterozygote-heterozygote base and the numbers of pubs of each genotype were determined [*number of pubs; observed*]. The percentage of each genotype from the total number of pubs is given in parentheses. The expected Mendelian distribution ratios [*number of pubs; expected*] and the p-value of the chi square test are given on the right. Note that for a total of 60 litters with 427 offspring of 15 heterozygous Tas1r1 breeding pairs, no significant deviation from the distribution predicted from Mendel's law was observed applying the chi square test (p≤0.05).

So far, our breeding experiments indicate that Tas1r1 deletion does not lead to severe impairment of reproduction. However, the lack of an apparent reproductive phenotype may be due to optimized laboratory breeding conditions, a phenomenon known to impede experimental studies in which gene knock-out animals were used to unravel regulatory mechanisms of reproduction [54], [55]. Alternatively, it is also conceivable that a yet unidentified subtype of class C G protein coupled receptors (GPCRs9 in male germ cells might be able to compensate the function of Tas1r1 in the Tas1r1/mCherry knock-in strain, as suggested previously for other GPCRs [56], [57]. Therefore, it was deemed necessary to perform morphological and functional studies of male reproductive organs and isolated spermatozoa of mutant animals. First, we looked for a possible effect on male reproductive organs by comparing total body and testis weight of adult Tas1r1 knock-out and wild-type mice. The data summarized in table 3 document that Tas1r1 deficiency did neither influence total body nor testis weight, and consequently the ratio of testis to body weight of mutant animals (0.73±0.03%) conformed to that of wild-type animals (0.75±0.03%).

**Table 3:** Effect of Tas1r1 deficiency on total body weight and weight of testes.

	Genotype		
	[+/+]	[+/−]	[−/−]
body weight [g]	28.0±0.7	28.2±0.4	27.2±0.5
testis weight [mg]	210±7	202±5	198±7
testis to body weight ratio [%]	0.75±0.03	0.72±0.02	0.73±0.03

Adult male homozygous ([*−/−*]), heterozygous ([*+/−*]) and wild-type animals ([*+/+*]) were analyzed for their total body and testis weight. Data represent mean values ± SEM of 17–46 animals of each genotype with no significant differences between Tas1r1-deficient mice and wild-type animals. Statistical analyses were performed using the Student's t-test. A p-value≤0.05 was considered to be statistically significant.

**Figure 5:**
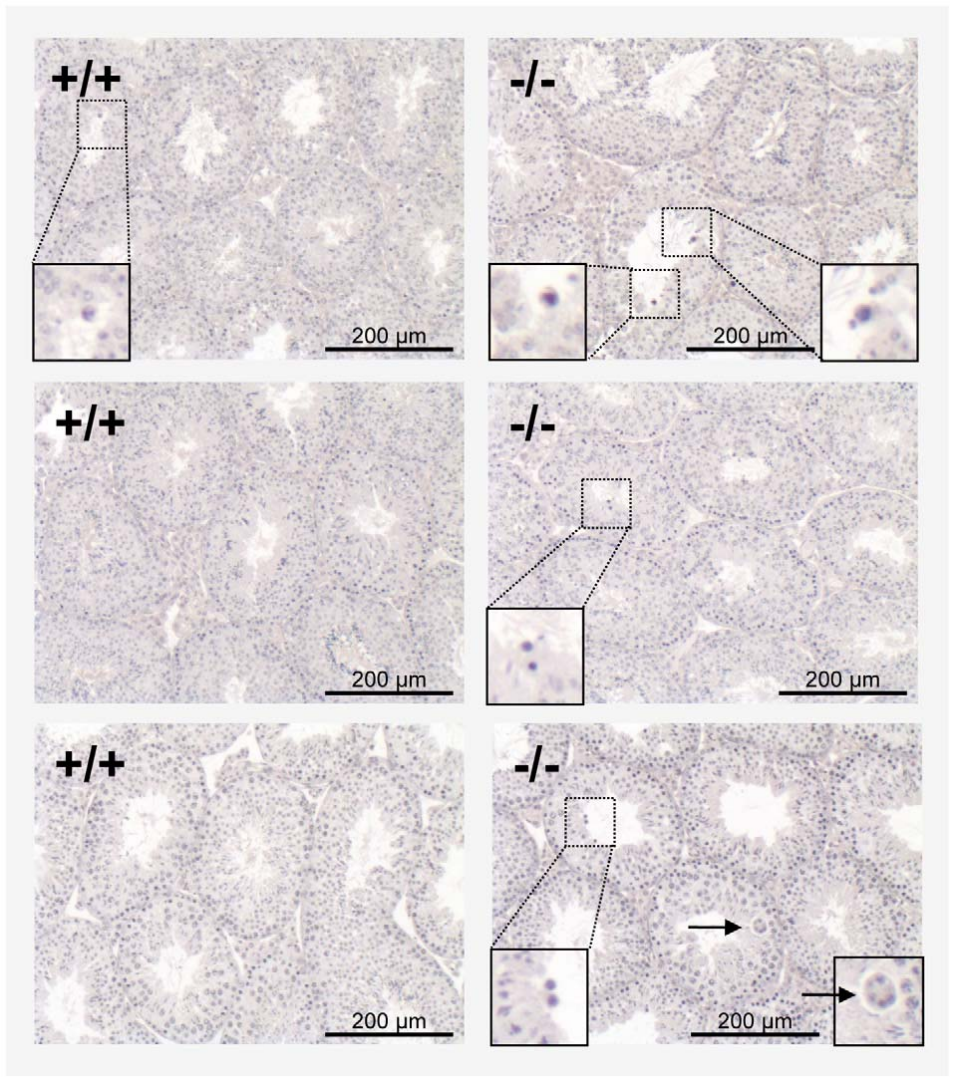
Morphological defects during spermatogenesis upon Tas1r1 gene deletion in Tas1r1/mCherry knock-in mice. Hematoxylin-Eosin stained sections of seminiferous tubules of wild-type and Tas1r1 knock out littermates were examined for abnormalities during spermatogenesis. Comparing testis of wild-type animals ([*+/+*]) and Tas1r1-deficient mice ([*−/−*]),Tas1r1 loss resulted in an increase in the number of spermatocytes which were abnormally found to be localized to the tubule's lumen instead of being concentrated to the basal cell layer (inserts with higher magnifications). In addition, some multinucleated giant cells were visible in single knock-out animals (lower right panel; arrowhead). The images are representatives of histological analyses of 4 adult Tas1r1 knock-out and wild-type littermate animals.

To assess whether Tas1r1 deletion alters testis morphology and/or germ cell proliferation, we examined the cellular organization of the seminiferous epithelium in Haematoxilin-Eosin (HE)-stained sections of Bouin-fixed and paraffin-embedded testes. Mice lacking Tas1r1 showed no apparent abnormalities in the size of their testes, and seminiferous tubules exhibited the full spectrum of ordered concentric layers of different developing germ cell populations (Fig. 5, right panels [*−/−*]). However, mild perturbations in the defined spatial organization of developing germ cell populations were observed. At higher magnification it becomes evident that in most of the mutant testes examined, miss-located spermatocytes were visible in the luminal part of the seminiferous tubules instead of a localization restricted to the more basal cell layers (Fig. 5; right panels; [*−/−*]); this miss-location was only rarely seen in wild-type animals (for representative s. Fig. 5; left panels; [*+/+*]). Moreover, we found multinucleated giant cells [58] in tubules of single Tas1r1 null mutant animals (Fig. 5; lower right panel; [*−/−*], arrow).

**Figure 6:**
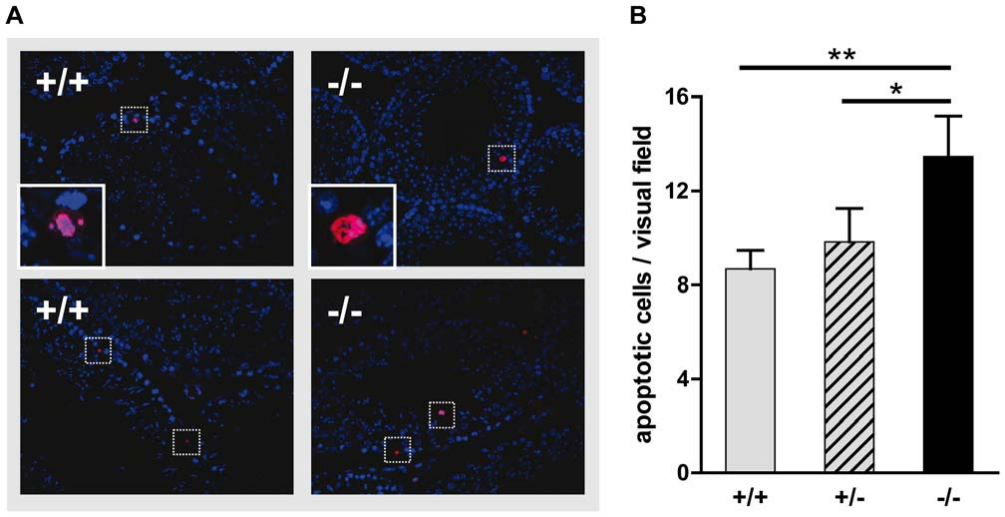
Determination of apoptotic cells in testicular sections of wild-type and Tas1r1/mCherry knock-in mice. [**A**] Paraffin sections of Bouin-fixed wild-type and Tas1r1-deficient testes were used in a fluorescent TUNEL assay and counterstained with DAPI to visualize nuclei and thus cellular compartmentalization. The two photomicrographs for each genotype document representative staining patterns of TUNEL positive cells of 5 male littermates per genotype. Note that in wild-type animals [*+/+*] as well as in Tas1r1-deficient mice [*−/−*], spatial localization of TUNEL-reactive cells (red) showed the usual accumulation within the basal cell layer of the testicular tubules. Moreover, apoptotic cells for each genotype did not show obvious differences in their morphology (higher magnifications presented in the inserts in the two upper panels). Micrographs are composed by an overlay of the two fluorescent channels (TUNEL, [red]; DAPI, [blue]); apoptotic TUNEL-positive cells are highlighted by insets. [**B**] Quantitative analysis of apoptotic cells in testes of wild-type, heterozygous and Tas1r1 null animals. Numbers of TUNEL-positive cells of the three genotypes are presented as apoptotic cells per visual field. Note that Tas1r1-deficient mice ([−/−]) show a significantly increased rate of apoptosis compared to wild-type ([*+/+*]) and heterozygous ([+/−]) animals. Data presented are mean values ± SEM; statistical analysis was done using a paired Student's t-test comparing apoptotic rates of corresponding littermates (*: p≤0.05; **: p<0.01). Testes of littermate animals (n = 5) of each genotype were analyzed, and sections were taken from two different regions. 3–4 tissue sections of each testicular domain were quantified for TUNEL positive germ cells counting 3–4 randomly chosen microscopic fields containing 25–30 seminiferous tubules each.

The quality of mature spermatozoa is usually assured by the described sequence of mitotic and meiotic divisions, but also by a regulated sorting of non-viable or genetically compromised germ cells, typically mediated by apoptotic selection during spermatogenesis [59], [60]. Since the impairment of DNA-repair in multinucleated cells leads to genetically defective germ cells [61], we examined whether the increase of giant and miss-localized cells in Tas1r1-deficient animals (Fig. 5; [*−/−*]) affects apoptosis during gem cell proliferation. Using the standardized TUNEL assay [62], we found that most of TUNEL-positive cells were normally localized to the basal cell layer of seminiferous tubules in littermates of both genotypes (Fig. 6; purple colored cells). However, quantification of the number of apoptotic germ cells per microscopic visual field which usually comprises 25–30 seminiferous tubules, revealed that apoptosis was significantly increased in Tas1r1 null-mutant mice (13.4±1.7 apoptotic cells per analyzed field; [*−/−*]; p = 0.003) value compared to wild-type animals (8.7±0.8 apoptotic cells/field; [*+/+*]) and Tas1r1 heterozygous mice (9.9±1.4 apoptotic cells/field [*+/−*]; p = 0.03); (Fig. 6B). This significant increase in apoptosis was also found by comparing the number of TUNEL positive cells per tubule in wild-type (0.33±0.02) and Tas1r1 null mutant males (0.45±0.04; p = 0.004), respectively.

**Figure 7:**
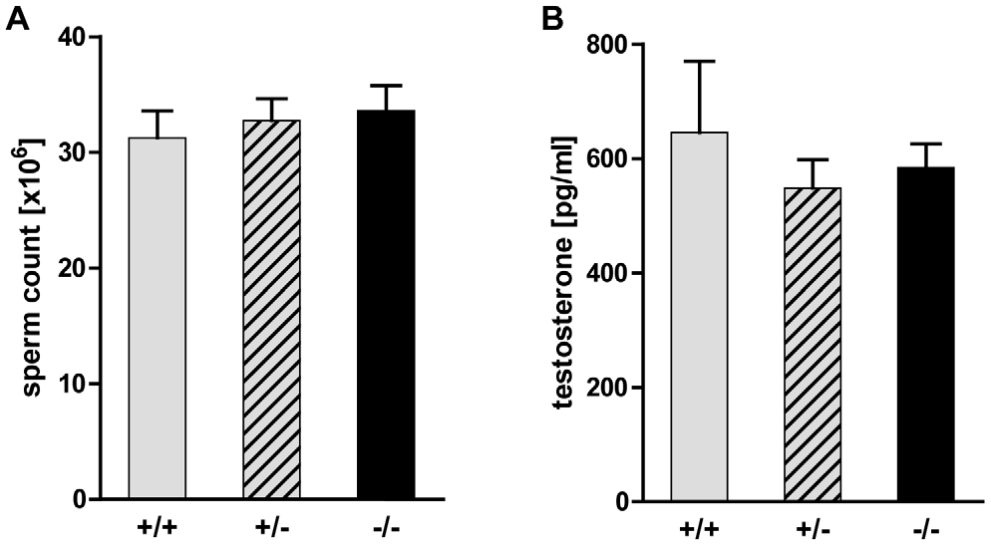
Sperm count and testosterone level of Tas1r1/mCherry knock-in mice. [**A**] Total number of caudal epididymal sperm in Tas1r1 null-mutant mice. Number of sperm in the caudal part of the epididymis were counted in male wild-type [*+/+*], heterozygous [*+/−*] and homozygous [*−/−*] mutantTas1r1 animals with identical strain background. Data are mean values ±SEM of 17–46 animals of the three genotypes. [**B**] Serum testosterone levels in Tas1r1-deficient male mice. Testosterone concentrations were measured in 4–6 month old male littermates of wild-type [*+/+*], heterozygous [*+/−*] and homozygous [*−/−*] Tas1r1 mice by a commercial enzyme-linked immunoassay. Data, expressed as means ± SEM, are obtained from 3 animals of each genotype with triplicate determinations; statistical analysis was done by a paired T*-*test; a p-value of ≤0.05 was considered to be statistically significant.

**Figure 8:**
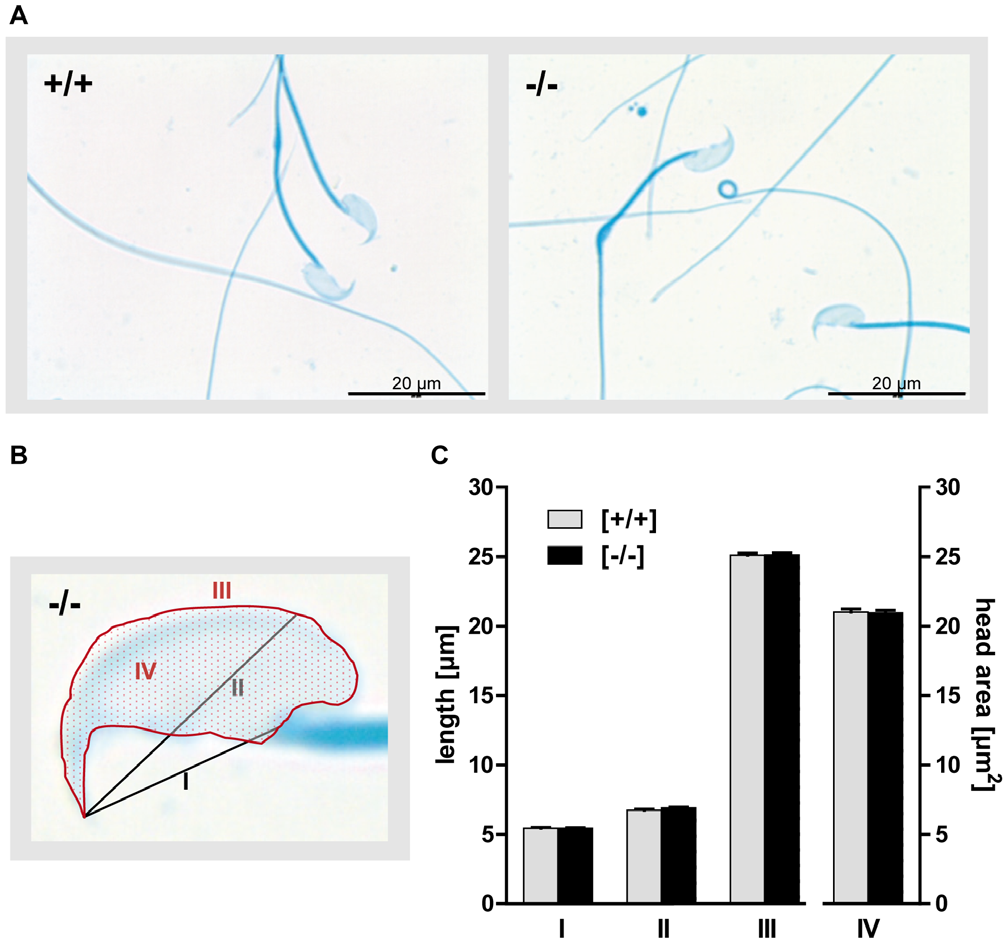
Morphology of Tas1r1-null sperm from the Tas1r1/mCherry mouse line. [**A**] Analysis of sperm morphology of wild-type and Tas1r1-deficient sperm. Isolated epidydymal sperm from C57BL/6 wild-type animals [*+/+*] and Tas1r1-deficient mice [−/−] were fixed, stained with Coomassie blue and subsequently subjected to bright field light microscopy. [**B and C**] Quantitative morphometric analysis of the sperm head of Tas1r1-deficient mice. To quantify dimensions of the sperm head, the length from the tip of the acrosome to the sperm neck ([*I*]) and to the post-acrosomal region ([*II*]) was scaled; in addition, circumference of sperm head ([*III*]) and the area of the whole sperm head ([*IV*]) were determined (for overview s. [**B**]). Data represent mean values ± SEM of the determined parameter which were obtained from 5 Tas1r1-deficient (black bars) and wild-type animals (grey bars); 8–15 sperm from each preparation were analyzed.

The observed increase in programmed cell death in Tas1r1-deficient mice did not lead to decreased testis weight (Table 3); however, disturbances in spermiogenesis (Fig. 5 and 6) could result in a reduced number of mature sperm cells and/or in non-functional spermatozoa. Therefore, we counted the number of mature spermatozoa isolated from the caudal part of the epididymis of Tas1r1/mCherry homozygous, heterozygous and wild-type male animals (Fig. 7A). In addition, the concentration of testosterone, essential for qualitatively and quantitatively normal spermatogenesis [63], [64], was analyzed in serum of the three genotypes (Fig. 7B). The total number of sperm obtained from the cauda epididymidis of all three genotypes was comparable (Fig. 7A); likewise, there was no difference in testosterone levels between wild-type and the two Tas1r1 genotypes (Fig. 7B). Moreover, examining germ cell morphology, we found that Tas1r1-deficient sperm do not exhibit obvious structural defects compared to wild-type sperm (Fig. 8A): Tas1r1 null sperm possess a normally formed flagellum and exhibit the characteristic hook-shaped outline of the head typical for mouse sperm. A quantitative morphometric analysis of the head (for parameters see Fig. 8B) confirmed this impression: Data summarized in figure 8C document that circumference and area of the sperm head were not different between the two genotypes ([*III*, *IV*]); similar results were obtained when measuring the length of the sperm head (Fig. 8C, [*I*]) and the distance between the proximal and distal ends of the acrosome (Fig. 8C; [*II*]).

### Physiology of Tas1r1-deficient spermatozoa

**Figure 9:**
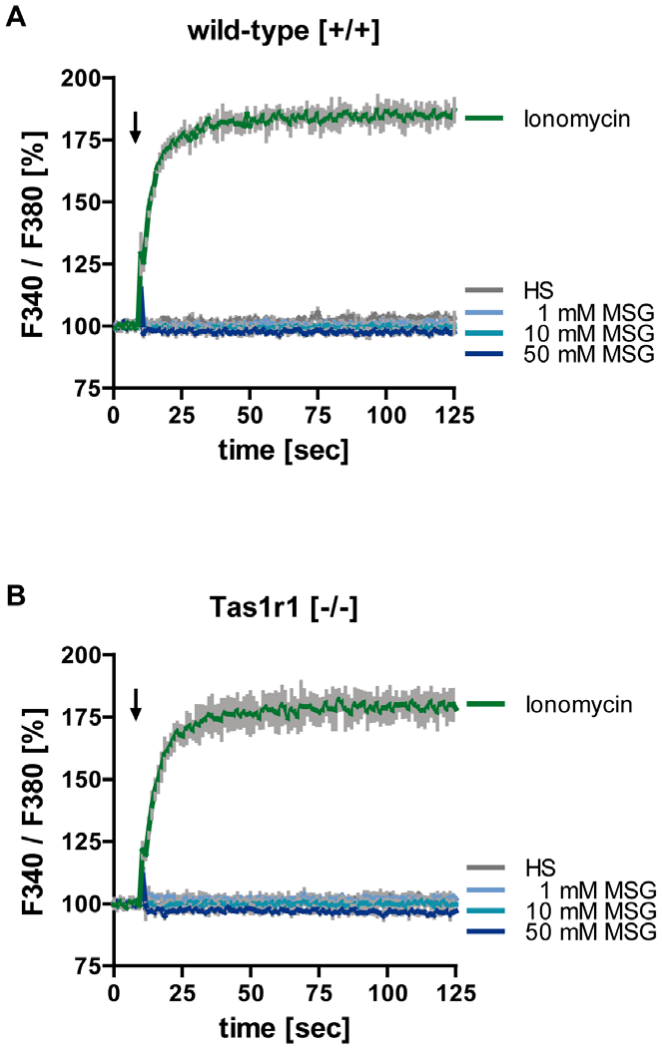
Effect of glutamate on intracellular calcium concentrations in wild-type and Tas1r1/mCherry knock-in mice. To evaluate the effect of MSG on intracellular Ca^2+^ concentration ([Ca^2+^]_i_) in sperm lacking the Tas1r1 receptor, capacitated cells were loaded with Fura-2/AM and subsequently fluorescence intensity of sperm populations was determined in a plate reader. Therefore, 90 µl of a capacitated sperm suspension (450,000–900,000 cells) were stimulated with different concentrations of MSG (1 mM MSG, 10 mM MSG, 50 mM MSG) by injecting 10 µl of a concentrated tastant stock solution. The concentration of the cation ionophore ionomycin used as a positive control was 5 µM; HS/NaHCO_3_ buffer alone served as negative control. Fura-*2* fluorescence was recorded with excitation wavelengths of 340 and 380 nm; subsequently data were calculated as ratio (*F340/F380*) and plotted against the time in seconds. Presented data are mean values ± SD of sperm of wild-type [*+/+*] and Tas1r1-deficient [*−/−*] mice measured in triplicates, which were representative for 3 experiments per genotype.

After excluding a severe morphological impairment of Tas1r1 null sperm (Fig. 8), we asked whether a physiological ligand of the Tas1r1/Tas1r3 dimer on the tongue, the amino acid monosodium glutamate (MSG), would be capable to activate Tas1rs in spermatozoa. Since changes of [Ca^2+^]i dynamics control critical sperm functions, like motility and pre-fusion processes such as chemotaxis and acrosome reaction [23], [65], [66], we monitored [Ca^2+^]i in response to MSG using the Fura-2 based ratiometric spectrometry. To assess dye loading and cell viability, each individual sperm preparation of the two genotypes was treated with the calcium ionophore ionomycin [67]. Figure 9 shows the normalized Fura-2 fluorescence ratio (F340/F380) of a sperm cell population as a function of time upon application of different concentrations of MSG (1 mM, 10 mM, 50 mM) which evoke robust [Ca^2+^]_i_ responses in taste cells of the tongue [43], [68]. Treatment of wild-type (Fig. 9A, [*+/*+]) and Tas1r1-deficient sperm (Fig. 9B, [*−/−*]) with ionomycin caused a similar increase in [Ca^2+^]_i_, in both genotypes, whereas MSG had no effect in the two sperm populations, even at high amino acid concentrations.

Next, we wondered whether the observed spermatogenic abnormalities (Fig. 5) and the increase in apoptosis during spermatogenesis (Fig. 6) may have any detrimental impact on physiological sperm function. Thus, sperm motility was evaluated. Table 4 summarizes standard motility parameters of spermatozoa isolated from wild-type [(*+/+*)], heterozygous [(*+/−*)] and Tas1r1/mCherry null mutant [(*−/−*)] littermates, determined by an automated CASA (computer-assisted motility analysis) setup. Quantifying different motility variables (left column, [*motility parameters*]), no statistical differences were detected between the three genotypes (s. [*p values*]), indicating that Tas1r1 deletion did not lead to a phenotypic difference in objective motility parameters.

**Table 4:** Motility analysis of wild-type and Tas1r1-deficent sperm.

	[+/+]	[+/−]		[−/−]	
motility parameter	mean ± SEM	mean ± SEM	p value	mean ± SEM	p value
Mot [%]	75.6±1.6	76.2±3.9	0.82	74.3±5.4	0.79
Prog [%]	37.4±11.0	37.8±11.6	0.94	36.0±11.3	0.63
VAP [µm/sec]	137.5±18.2	137.1±14.4	0.96	135.3±18.5	0.82
VSL [µm/sec]	90.2±17.1	89.2±14.0	0.89	88.5±16.1	0.82
VCL [µm/sec]	286.7±24.1	281.2±22.6	0.64	276.3±26.5	0.55
ALH [µm]	14.4±0.3	14.7±0.1	0.56	14.6±0.7	0.75
BCF [Hz]	33.1±4.1	33.7±3.4	0.80	33.6±3.7	0.56
STR [%]	62.5±4.4	62.0±4.5	0.75	62.6±3.9	0.83
LIN [%]	31.8±3.2	32.0±2.6	0.85	32.6±2.8	0.23

Computer-assisted sperm analysis (CASA) was performed using an IVOS sperm analyzer (Hamilton Thorne, Berverly, USA). Parameters analyzed are given on the left. Motility values of wild-type [*+/+*] and Tas1r1 heterozygous [*+/−*] and homozygous [*−/−*] sperm are shown as mean values ± SEM of 3 littermate animals for each genotype. Additionally, p-values of a paired Student's T-Test [*p values*] are given. The following parameters are shown: Percentage of motile sperm [*Mot*], percentage of sperm with active motility [*Prog*], averaged path velocity [*VAP*], straight line velocity [*VSL*], curvilinear velocity [*VCL*], amplitude of lateral head displacement [*ALH*], beat cross frequency [*BCF*], straightness [*STR*], linearity [*LIN*]. A minimum of 2000 spermatozoa was analyzed per animal. Note that wild-type sperm and Tas1r1-deficient spermatozoa did not show any significant differences (p-value≤0.05) in the analyzed motility parameters.

**Figure 10:**
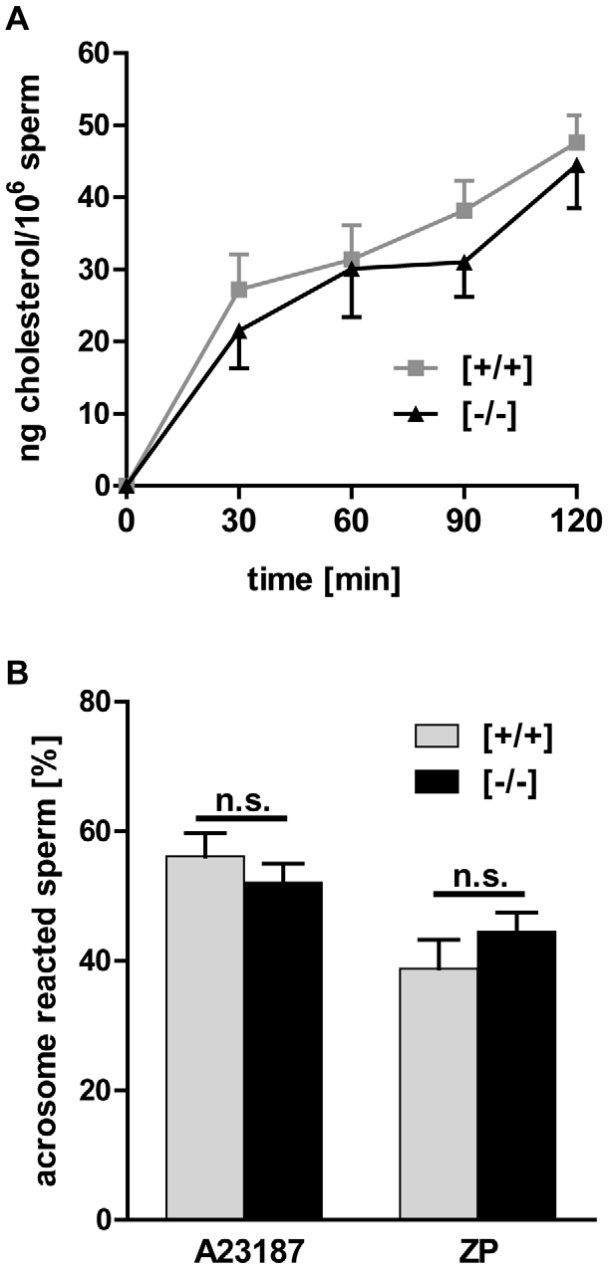
Capacitation and acrosome reaction in Tas1r1 null sperm from the Tas1r1/mCherry mouse line. [**A**] Capacitation dependent efflux of cholesterol in Tas1r1-deficient mice. To quantify capacitation dependent cholesterol release in isolated epididymal sperm of wild-type and Tas1r1 null mutant animals, equal amounts of a homogeneous sperm suspension were incubated for different time periods (0 min, 30 min, 60 min, 90 min, 120 min) in HS/BSA/NaHCO_3_ as described in Materials and Methods. At the indicated time points, aliquots of the supernatant were collected and used to measure cholesterol release using a fluorometric-based quantification kit. Obtained data were calculated as cholesterol efflux per cell after subtracting basal cholesterol content at the beginning of the incubation (0 min: [*+/+*]: 42±3 ng cholesterol/10^6^ sperm; [*−/−*]: 37±2 ng cholesterol/10^6^ sperm). Time-dependent sterol release in sperm of both genotypes increased over time and showed no significant difference (p≤0.05). Data, presented as mean values ± SEM, are the average of nine independent sperm preparations of C57BL/6wild-types and Tas1r1-deficient animals from the same colony. [**B**] A23187 and *zona pellucida* induced acrosomal secretion in Tas1r1 null sperm. To assess whether Tas1r1-deficient sperm show a defect in the acrosomal exocytotic machinery or in recognizing the egg's coat, respectively, *in vitro* capacitated spermatozoa of wild-type and Tas1r1 null mutant littermates were either treated with 10 µM A23187 [*A23187*] or alternatively with solubilised *zona pellucida* [*ZP*] at 37°C for 30 min. Subsequently, aliquots of sperm were stained with Commassie blue G.250 and acrosomal status was quantified by counting at least 200 cells for each condition. Data, calculated as absolute percentages of acrosome reacted sperm represent mean values ± SEM of independent experiments with different mouse sperm preparations ([*A23187*], n = 15; [*ZP*], n = 7). Spontaneously occurring secretion rates were determined incubating sperm in corresponding buffer used to dilute the stimulating compounds [buffer with DMSO: wild-type [*+/+*]: 28.1±2.2%; Tas1r1 [*−/−*]: 35.2±2.5%; ZP buffer alone: wild-type [*+/+*]: 33.1±3.5%; Tas1r1 [*−/−*]: 37.7±3.0%). Statistical analysis was done using a Student's t-test comparing acrosome reacted sperm of both genotypes.

To assess whether Tas1r1 deficiency affects sperm acrosome reaction, loss of the acrosomal vesicle was quantified in sperm of littermates of both genotypes. Physiological acrosome reaction, which can only occur in fully capacitated spermatozoa [55], [69], is accompanied by characteristic lipid redistributions and an efflux of cholesterol from the plasma membrane, subsequently affecting membrane-associated signaling processes [70], [71], [72], [73]. Since Tas1r1 is a member of the superfamily of heptahelical GPCRs [74], we initially evaluated a potential effect of Tas1r1 gene inactivation on sperm capacitation; hence, sterol efflux of epididymal sperm collected from wild-type and Tas1r1-deficient mice was quantified by incubating sperm in an *in vitro* capacitation medium for different time periods [75]. Figure 10A illustrates that sperm of both genotypes show a steady and consistent cholesterol efflux over time, with no significant difference between wild-type and Tas1r1 null spermatozoa. To test whether Tas1r1 gene deletion would hamper acrosomal secretion, the effect of directly increasing [Ca^2+^]i by the Ca^2+^ ionophore A23187 [76] was assessed. A23187-elicited increases in [Ca^2+^]_i_ bypass *zona pellucida*-mediated activation of signal transduction pathway/s [77], and thus allow to evaluate the exocytotic fusion apparatus. Caudal epididymal sperm of wild-type and Tas1r1 littermates were stimulated with 10 µM of A23187 [78] or with the corresponding control buffer (0.1% dimethyl sulfoxide [DMSO]) and subsequently the proportion of acrosome-intact spermatozoa was determined. Figure 10B (left column pair; [*A23187*]) illustrates that A23187 markedly elevated acrosomal secretion rates in sperm of both genotypes when compared to the basic level of spontaneously acrosome-reacted spermatozoa ([*+/+*]: 28.1±2.2%; [−/−]: 35.2±2.5%). However, there was no significant difference in the incidence of acrosomal loss between wild-type and Tas1r1-deficient sperm indicating that the acrosomal machinery in Tas1r1-deficient cells is intact. The physiological ligand for triggering acrosome reaction is the *zona pellucida* of the mature oocyte [79]. To clarify whether Tas1r1 in spermatozoa is directly involved in zona recognition and subsequent induction of acrosomal exocytosis, we treated capacitated epididymal sperm of wild-type and Tas1r1 knock-out littermates with isolated and solubilized *zona pellucida*, and subsequently germ cells were quantified for acrosome intact spermatozoa. A significant induction of acrosome reaction was observed for sperm of Tas1r1-deficient animals treated with *zona pellucida* (Fig. 10B; left column pair, [*ZP*]). Moreover, ZP-evoked acrosome reaction was not significantly different between sperm of both genotypes, hence indicating that binding to *zona pellucida* and activation of coupled intracellular signaling cascade/s [69] was not influenced upon Tas1r1 deletion.

**Figure 11:**
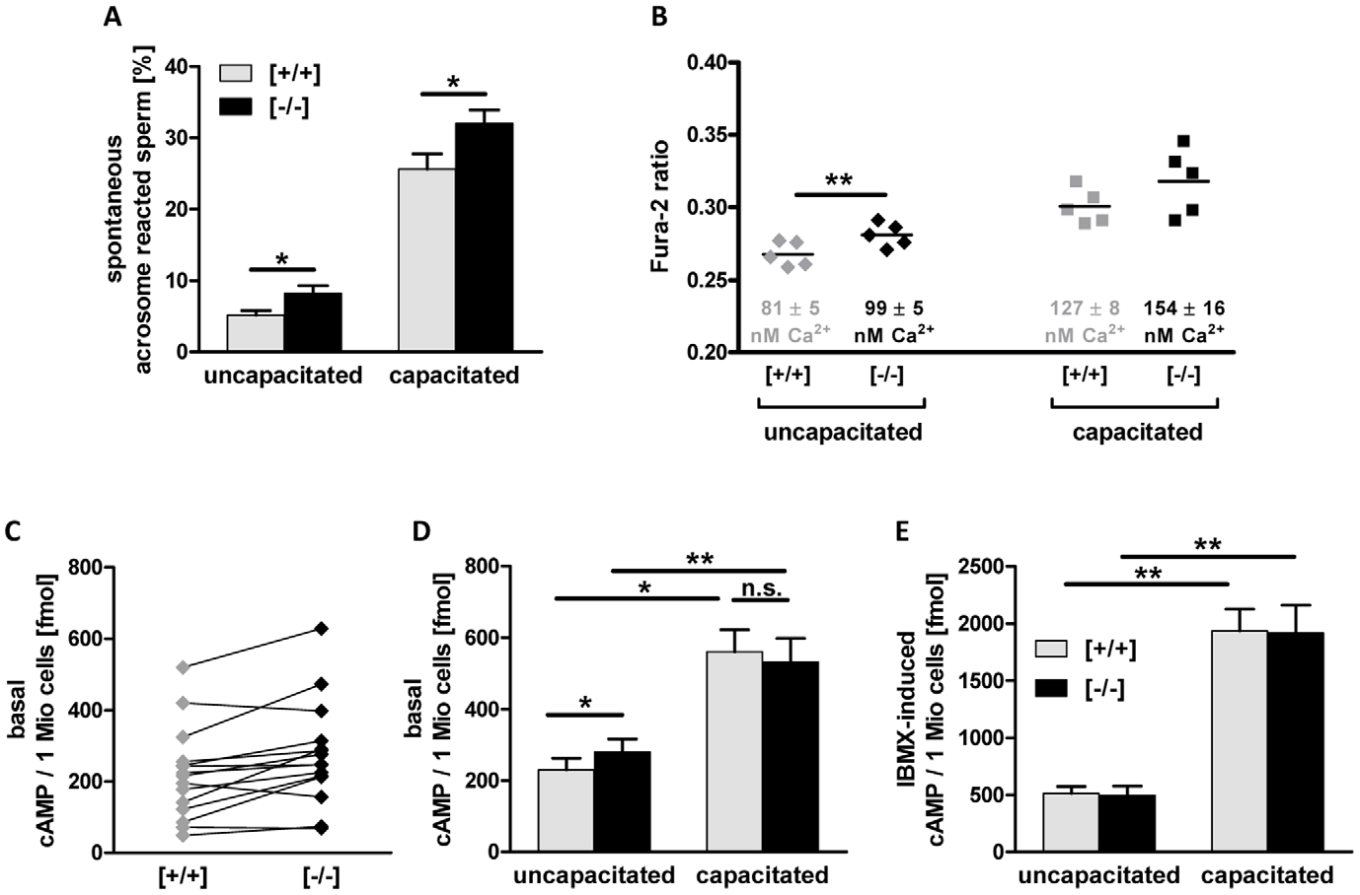
Tas1r1 deletion results in increased spontaneous acrosome reaction and elevated cytosolic Ca^2+^ and cAMP levels. [**A**] Incidence of spontaneous loss of the acrosomal vesicle in sperm from Tas1r1 knock-out mice compared to control wild-type sperm. To quantify spontaneous acrosome reaction of uncapacitated and fully capacitated sperm, epididymal spermatozoa of wild-type and Tas1r1 null mutant mice with identical genetic background were either directly assessed for acrosomal secretion rates or incubated for 90 min in capacitation medium (HS/BSA/NaHCO_3_). Data shown are mean values ± SEM of 15 independent experiments of different mouse sperm preparations. Obtained data were subjected to a Student's t-test for determination of significant differences (*: p≤0.05) between pairs of both genotypes. [**B**] Comparison of [Ca^2+^]_i_, of wild-type and Tas1r1-deficient spermatozoa. To determine basal [Ca^2+^]_i_ in the head region of wild-type ([*+/+*], grey rhombs and squares) and Tas1r1-deficient ([*−/−*], black rhombs and squares) spermatozoa, epididymal sperm cells were either directly loaded with Fura-2AM ([*uncapacitated*], rhombs on the left side), or capacitated for 60 min prior Fura-2 loading ([*capacitated*], squares on the right side). Subsequently, Fura-2 fluorescence at 510 nm was measured at excitation wavelengths of 340 and 380 nm using a microscope based imaging system (TillPhotonics, Graefelfing, Germany). Fura-2 ratios (F340/F380) were determined for at least 14 cells per sperm preparation (total number of measured sperm cells: uncapacitated: 151 [*+/+*], 136 [*−/−*]); capacitated sperm: 168 [*+/+*], 181 [*−/−*]). [Ca^2+^]_i_ was calculated using the mean Fura-2 ratio of each animal (*F340/F380*) according to [84]. Only spermatozoa that showed [Ca^2+^]_i_, increases upon stimulation with the calcium ionophore ionomycin were considered. Shown are vertical scatter plots of Fura-2 ratios of isolated spermatozoa of 5 animals for each genotype (littermates and animals with matched genetic background); the mean Fura-2 ratio is indicated by a bar. Mean values ± SEM of calculated [Ca^2+^]_i_, for each genotype are given in numbers in the lower part of the graph.Statistical analyses were done using a paired Student's t-test (**: p<0.01). [**C**] Vertical scatter plot of basal cAMP concentration in uncapacitated spermatozoa. Shown are basal cAMP concentrations of epididymal sperm isolated in HS buffer. Littermate animals and animals with identical genetic background were prepared and assayed in parallel. cAMP values of corresponding animal pairs are connected by a line. Note that in 13 of 15 analyzed animal pairs, cAMP concentrations were higher in Tas1r1 -deficient [*−/−*] mice than in wild-type [*+/+*] animals. [**D–E**] cAMP concentrations in Tas1r1-deficient sperm compared to sperm of wild-type animals. Epididymal sperm of wild-type [*+/+*] and Tas1r1-deficient [*−/−*] mice were either isolated in HS (for 15 min) [*uncapacitated*] or in capacitation buffer (HS/BSA/NaHCO_3_ for 60 min; [*capacitated*]), and subsequently treated for 5 min at 37°C with buffer alone [**D**] (uncapacitated: n = 15; capacitated: n = 11) or with 0.5 mM IMBX [**E**] (uncapacitated: n = 13; capacitated: n = 9). After shock-freezing the cells in liquid nitrogen, cAMP was extracted with PCA (7%), and quantified using a commercially available EIA kit. Data are mean values ± SEM. Sperm of littermate animals and animals with identical genetic background and age were assayed in parallel and compared using a paired student's T-Test (*: p≤0.05; **: p<0.01).

When quantifying acrosomal secretion rates in response to different stimuli (zona pellucida, [Fig. 10B]; MSG, [Fig. S3A]; sweet tastants, [glucose, saccharin, acesulfam K, thaumatin], [Fig. S3B]), we always noted a slightly higher percentage of acrosome-reacted spermatozoa inTas1r1 null than wild-type sperm. When acrosome reaction was artificially induced by the Ca^2+^ ionophore, no such increased acrosome reaction was observed in TasR1-deficient sperm (Fig. 10B; left columns; [*A23187*]). Since this elevated proportion of acrosome-reacted sperm in Tas1r1 null mice may be based on a constantly higher spontaneous rate of acrosomal exocytosis, we quantified the acrosomal status of uncapacitated and fully capacitated spermatozoa of untreated wild-type and Tas1r1 null sperm. The results summarized in figure 11A (left column pair) show that freshly isolated sperm [*uncapacitated*] show a significantly higher rate of spontaneous loss of the acrosomal vesicle compared to wild-type sperm (p<0.05). The same significant increase in the incidence of spontaneous acrosome reaction was detected for capacitated Tas1r1 [−/−] sperm (Fig. 11A; right column pair),

Since Ca^2+^ and the second messenger cyclic adenosine monophosphate (cAMP) are both associated with the generation of umami-dependent cellular responses in taste cells of the tongue (for review see [45]) and Ca^2+^ as well as cAMP have also been described as key regulators of the acrosome reaction (for review see [69], [80], [81]), we examined free cytosolic Ca^2+^ [Ca^2+^]_i_ (Fig. 11B) and cAMP concentrations (Fig. 11C and D) in spermatozoa of Tas1r1-deficient animals and sperm of wild-type littermates. To quantify [Ca^2+^]_I_, isolated caudal epididymal spermatozoa of both genotypes were loaded with the calcium indicator Fura-2, either directly or after capacitation, and subsequently the F340/F380 ratio in the sperm head was determined. Figure 11B represents a scatter plot of mean values of Fura-2 ratios of sperm of 5 animals per genotype (at least 14 cells per animal) for uncapacitated (left pair) and capacitated cells (right pair).

As described previously ([82], [83], for review see also [80]), Fura-2 ratios of capacitated sperm [capacitated] were clearly higher than in uncapacitated spermatozoa [uncapacitated], independent of the analyzed genotype. Calculating [Ca^2+^]_i_ according to [84], it becomes obvious that uncapacitated as well as *in vitro* capacitated sperm of Tas1r1-deficient animals are characterized by a higher [Ca^2+^]_i_ than sperm of wild-type animals (Fig. 11B; uncapacitated: [+/+]: 81±5 nM, [−/−]: 99±5 nM; capacitated: [+/+]: 127±8 nM, [−/−]: 154±16 nM), with uncapacitated sperm showing a significant difference between both genotypes (p<0.01).

Analyzing basal cAMP levels in uncapacitated sperm of wild-type and Tas1r1-deficient animals, a comparable difference in intracellular messenger concentration was observed (Fig. 11C): Uncapacitated spermatozoa of wild-type animals had a basal intracellular cAMP concentration of 230±33 fmol/10^6^ sperm consistent with previous measurements of cAMP levels in mouse sperm [85]. Although basal cAMP content varied broadly between different animals (see scatter plot, Fig. 11C), in almost all of the analyzed mouse pairs (13 out of 15), the cAMP content of uncapacitated cells of Tas1r1 deficient animals was higher than in corresponding wild-type mice (for a detailed overview of absolute basal cAMP values determined for each animal pair; [Table S1]). This tendency towards higher cAMP levels in the gene-deficient animals led to a significantly increased mean basal cAMP concentration in uncapacitated Tas1r1 deficient sperm (280±36 fmol/10^6^ sperm, paired t-test of animals of both genotypes with identical genetic background; p<0.05). However, when comparing basal cAMP concentrations in capacitated sperm, the difference in cAMP between the two genotypes was no longer significant (Fig. 11D, right column pair): In addition to the expected increase in cAMP levels detected upon capacitation [86], most probably caused by activation of soluble adenylate cyclase (sAC) by bicarbonate [87] and/or Ca^2+^ [88] in the capacitation buffer, cAMP concentrations in wild-type (560±62 fmol/10^6^sperm) and Tas1r1-deficient sperm were almost identical (530±66 fmol/10^6^sperm). Since differences in cAMP levels in spermatozoa of mutant animals might be due to altered second messenger production or alternatively enhanced catabolic activity, the effect of 3-isobutyl-1-methylxanthine (IBMX), a phosphodiesterase (PDE) blocker [89], was analyzed (Fig. 11E). IBMX treatment of uncapacitated spermatozoa led to a strong and significant (p<0.01) accumulation of cAMP in wild-type (513±60 fmol/10^6^ sperm) as well as Tas1r1-deficient sperm (495±82 fmol/10^6^ sperm) compared to basal cAMP levels in the two immature sperm populations (Fig. 11E, [*uncapacitated*]); similar results were obtained when comparing cAMP levels in capacitated sperm ([*+/+*]: 1937±190 fmol/10^6^ sperm; [*−/−*]: 1916±244). However, no significant difference in overall cAMP accumulation was detectable when hydrolysis was blocked by IBMX (Fig. 11E) or by the selective PDE 4 inhibitor rolipram (Table S2), indicating that the increase in cAMP levels in sperm of Tas1r1-deficient animals might be caused by differences in PDE-dependent cAMP degradation.

## Discussion

### 1. Extra-oral expression of chemosensory receptors

Although it is generally assumed that sperm possess chemosensory abilities to respond to the multitude of environmental cues in the female reproductive tract during their transit towards the egg, the sperm's repertoire of potential chemosensory receptor proteins is largely unknown. The present manuscript describes for the first time the expression of taste receptors in mammalian spermatozoa and provides evidence for the presence of the two subunits of the umami taste receptor (Tas1r1/Tas1r3) in mouse and human sperm, while transcripts for the sweet taste receptor (Tas1r2) were not detected. This observation of extra-oral expression of taste receptor proteins not only fits well with the concept of a widespread taste receptor expression profile in non-taste tissues (for review see [36]) and thus a non-gustatory function, but in addition indicates that sperm, besides olfactory receptors, appear to utilize another phylogenetically ancient sensory modality to scan their external chemical environment. Ligand binding of class C GPCRs is mediated by a Venus flytrap-like module, formed by the dimerization of their long N-terminal regions [90], whereas odorants receptors bind ligands within their 7 transmembrane domains (for review see [91]). Thus, one may hypothesize that the two chemosensory receptor families identified in mammalian sperm are specialized to recognize chemically disparate compounds dissolved in the fluids of the female reproductive tract.

### 2. Dimerization/Ligands

**Figure 12:**
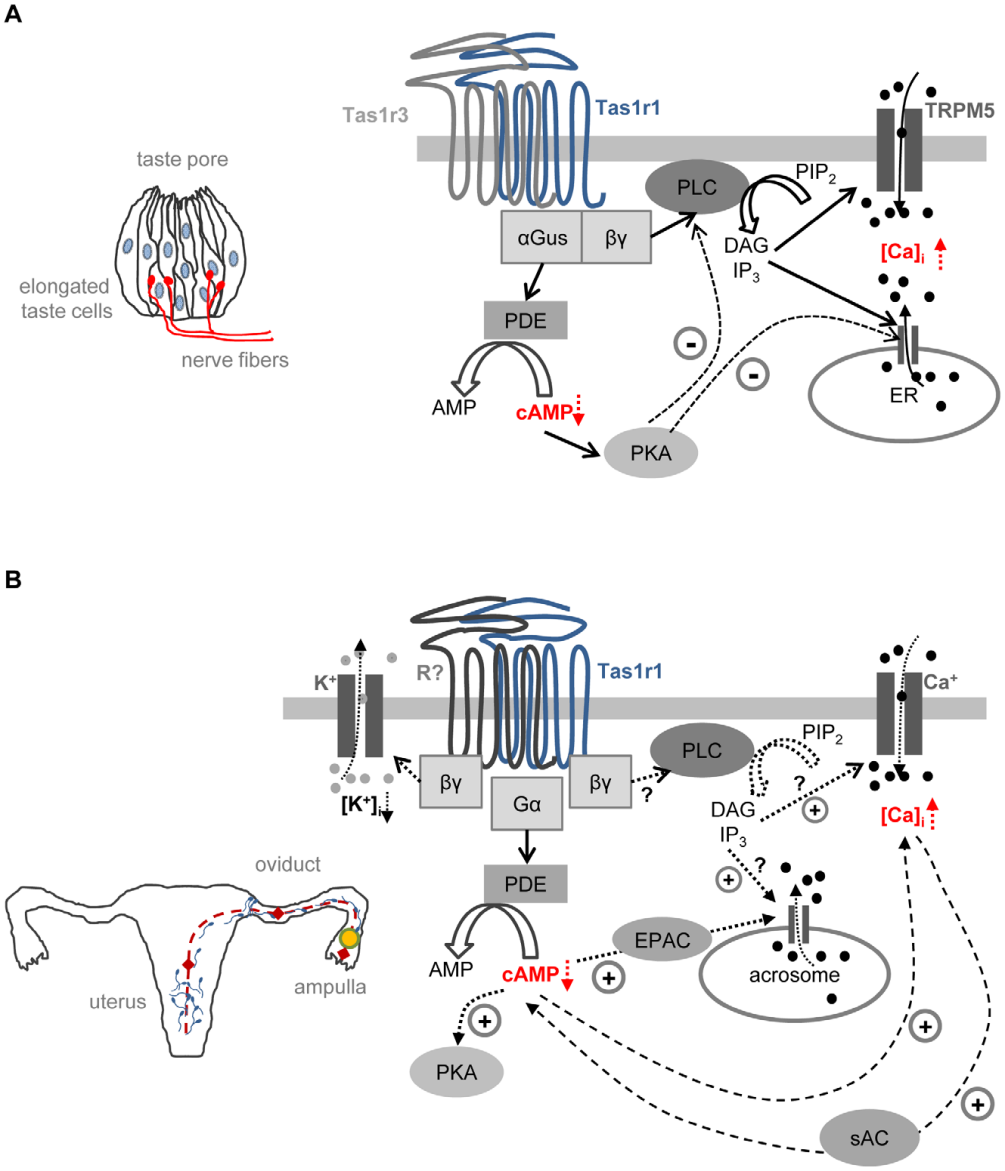
Working model illustrating a possible functional role of taste receptor signaling in taste cells and spermatozoa. [**A**] Model for the transduction cascade of the umami receptor in taste cells. On the left, a schematic drawing of the onion-like structure of a single taste bud formed by elongated taste cells is shown. The peripheral ends of the 50–100 taste cells in one taste bud terminate at the gustatory pore; taste information is coded by afferent nerve fibers which innervate the taste buds and come close to type II receptor cells but only form conventional chemical synapses with the basolateral membrane of type III taste cells. In taste cells, the Tas1r1 and Tas1r3 receptors form a functional dimer which is able to recognize amino acids such as MSG. Upon ligand binding, the umami receptor activates a trimeric G Protein consisting of α-gustducin [*αGus*] and β_3_ and γ_13_ [*βγ*]. The βγ subunit activates phopholipase Cβ_2_ [*PLC*] which cleaves phosphatidylinositol 4, 5-bisphosphate [*PIP_2_*] to inositol trisphoshate [*IP_3_*] and diacylglycerol [*DAG*]. IP_3_ mediates an increase in intracellular calcium by activation of calcium channels in the endoplasmic reticulum [*ER*] and subsequently an influx of calcium through ion channels in the plasma membrane [*TRPM5*]. Simultaneously, released α-gustducin can activate phosphodiesterase, resulting in a decrease of intracellular levels of cyclic adenosine monophosphate [*cAMP*]. A crosstalk between the two pathways exists through a cAMP regulated activation of protein kinas A [*PKA*] which inhibits PLC and the IP_3_-receptor in the ER. This mechanism may ensure adequate Ca^2+^ signaling to taste stimuli by keeping the taste cell in a tonically suppressed state. The drawing was modified from Ref. [45] and [109]. [**B**] Putative model of Tas1 taste receptor signaling in spermatozoa. The schematic drawing in the left signifies the sperm's journey in the different sections of the female genital tract [*uterus*, *oviduct*, *ampulla*] which sperm have to transit to reach the egg in the ampullar region of the oviduct (dotted red line). In sperm cells, the Tas1r1 protein [*Tas1r1*] may dimerize with its taste partner Tas1r3 or with a yet not identified receptor [*R?*]. G protein activation results in the release of a G protein α-subunit [*Gα*] which activates phosphodiesterase [*PDE*], thus leading to the hydrolysis of cAMP. In this model, an activation of the receptor dimer [*Tas1r1/R?*] by chemosensory ligands within the different regions of the female genital tract (red rhoms) or a constitutively active receptor may ensure low cAMP levels, thereby preventing cAMP-triggered maturation processes of the sperm, like capacitation, motility or acrosome reaction, before the sperm reaches the egg in the ampullary part of the oviduct. If the simultaneously released Gβγ complex [*βγ*] indeed stimulates PLC in analogy to taste cells or alternatively activates potassium [K^+^] channels in sperm, is currently not clear. Constant cAMP hydrolysis can be overcome during sperm maturation either by an decrease in taste receptor activation controlled by changes in the composition of chemical components in the different fluids of the female genital tract or by an increase in [Ca^2+^]_i_, or high bicarbonate concentration which would lead to an activation of the soluble adenylatecyclase [*sAC*] in spermatozoa. For seek of simplicity, regulatory effects of PKA activation or EPAC stimulation on calcium channels or the IP_3_ receptor are omitted in the model.

Our results indicate that the onset of Tas1r3 expression resembles that of Tas1r1 during spermatogenesis (Fig. 3), and that their sub-cellular localization in mature spermatozoa is comparable (Fig. 4; Fig. S2). However, we currently cannot definitively affirm that the observed co-localization indeed results in a physical interaction of the tongue-specific dimerization partners to constitute a functional receptor complex in male germ cells. Given that sperm provide a unique response spectrum which obviously does not include [Ca^2+^]_i_ responses to MSG (Fig. 9) one might suggest that taste receptors in sperm form functional hetero-dimers or even larger hetero-oligomers [92] which are different from the ones in taste buds of the tongue and which may also show some compensatory effect upon Tas1r1 deletion, thus providing an explanation for the inconspicuous reproductive phenotype of Tas1r1 (*−/−*) animals. Since hetero-dimerization between distinct GPCRs was described to be responsible for the generation of pharmacologically defined receptors with a unique mode of activation (e. g. agonist affinity, efficacy, signaling properties, positive or negative allosteric modulation) [90], [93], one may speculate that such a “new” receptor entity which had also been proposed for gastric endocrine cells [94] might specifically recognize endogenous reproductive agonists. This assumption seems even more attractive considering that the Tas1r1/Tas1r3 dimer mediating umami taste sensation on the tongue is potentiated by purine nucleotides, like inosine monophosphate (IMP) [95], a “fine tuning” mechanism, which for taste receptors in spermatozoa may be realized by reproductive specific allosteric modulators. In this context, it is interesting to mention that glutamate concentrations in the female genital tract are high in the uterus und decline constantly on the way to the egg in the ampullary region of the follicular tube (s. Model, Fig. 12B), whereas all other amino acids show their highest concentration in the oviductal region [3], thus indicating that distinct gradients of potential taste receptor ligands indeed exist within the different compartments of the female genital tract. Thus, sperm may sense increments of such chemical compounds on their way to the mature egg in the ampullary part of the fallopian tube. However, at present we cannot definitively decide whether MSG can induce cAMP signals in Tas1R1 mutant sperm due to elevated basal cAMP levels in uncapacitated Tas1r1 null sperm (s. Fig. 11C/D and Fig. S4). Together with the observation that MSG did not elicit an increase in [Ca^2+^]_i_ (Fig. 9), and that MSG was ineffective in inducing acrosome reaction in spermatozoa (Fig. S3), it still remains debatable whether glutamate is indeed an active ligand of the Tas1r1 in spermatozoa.

However, with regard to the most prominent dimerization candidates of class C GPCRs [96], [97], i. e. metabotropic glutafomate

### 3. Signaling and function of taste receptors in spermatozoa

The observed expression of taste receptors in mammalian spermatozoa is consistent with the recent finding that the taste G protein α-gustducin is also present in mammalian spermatozoa [38]. However, using subtype-specific antisera for signaling molecules involved in the transduction of sweet, bitter and umami taste in taste buds [103], like Gβ_3_ [104], Gγ_13_ [105] and PLCβ2 [103], [106], we found that these downstream signaling components were not unambiguously detectable in spermatozoa (data not shown). However, taste transduction comprises Gβ_3_γ_13_-mediated PLCβ2-induced generation of DAG and IP_3_ (Inositol 1,4,5-trisphosphate) as well as a simultaneous change of cAMP levels [107], [108], [109] (Fig. 12A). In particular a role of cAMP is notable, since α-gustducin (−/−) mice have been found to exhibit elevated basal cAMP levels in taste buds which might be due to a lack of constant PDE activation through α-gustducin [109] (s. Fig. 12A). Measuring cAMP concentrations in uncapacitated sperm of Tas1r1/mCherry knock-in animals, we also observed elevated basal cAMP levels compared to wild-type sperm (Fig. 11C and D), whereas upon PDE inhibition (Fig. 11E) or capacitation (Fig. 11D, right column pair) this difference was adjusted. Many GPCRs display a certain constitutive activity [110] which appears to be responsible for the sweet taste of pure water in taste buds of the tongue [111]. Thus, it is conceivable that taste receptors in spermatozoa may also be constitutively active, resulting in lower cAMP levels in wild-type spermatozoa (Fig. 11D).

However, the main question concerns the physiological relevance of low cAMP levels for spermatozoa mediated by taste receptor activation. In taste cells, it has been suggested that cAMP antagonizes responses to umami stimuli by modulating the sensitivity of the PLC signaling pathway [45], [109], probably by a PKA mediated phosphorylation and thus inhibition of PLCβ2 and the IP_3_-R [109] (Fig. 12A). Because cAMP and PKA are known to be key regulators of capacitation and of sperm motility as well as acrosome reaction (for review see [69], [112], [113], [114]) and because capacitated sperm exhibit much higher cAMP concentrations irrespective of Tas1r1 expression (Fig. 11C, right column pair), one may speculate that in uncapacitated sperm [115] taste receptors are permanently activated by chemical compounds dissolved in the aqueous environment of the female reproductive tract which might result in tonic suppression of cAMP levels. In analogy to the taste system, this effect might be mediated by G Protein α-subunit-controlled PDE stimulation (s. model in Fig. 12B). However, upon reaching the isthmus of the oviduct, bicarbonate and Ca^2+^ stimulation of the sAC [87], [88] may overcome PDE-catalyzed cAMP hydrolysis, thus resulting in cAMP accumulation and thereby complete maturation of the germ cell. Although it is currently not clear which target signaling molecules might be affected by the simultaneously released Gβγ complex (Fig. 12B), such a mechanism would prevent unintended acrosome reactions which may otherwise be triggered by cAMP- or PKA-controlled activation of Ca^2+^ channels [116], [117], [118], [119] or the recently described EPAC (exchange factor directly activated by cAMP) signaling pathway [120]. Thus, elevated intracellular pre-capacitatory cAMP levels of Tas1r1 null sperm are fully compatible with the observed increase in basal [Ca^2+^]_i_ of Tas1r1-deficient sperm (Fig. 11B) and the significantly higher level of spontaneous acrosome reaction (Fig. 11A). Although we currently cannot exclude that the increase in apoptosis seen in Tas1r1 testes (Fig. 6) is due to deleterious effects of the cloning cassette used to generate the mutant animals or the fluorescent protein itself, adaptive mechanisms might exist which could compensate for the higher rate of apoptosis [121], [122], thus leading to the mild phenotype noted for the Tas1r1 knockout animals. Tas1r1-deletion may also lead to higher cAMP concentrations during spermatogenesis, especially because male germ cell development is known to be supported by PKA activation [123]. Future studies will have to address the issue whether Tas1r1 deletion also leads to elevated cAMP levels in other tissues expressing taste receptor proteins in order to understand possible non-gustatory functions of this receptor family. For the reproductive system, the ultimate challenge is to identify additional sensory GPCRs expressed in germ cells and to reveal which sperm-specific heterodimers of taste receptors might be involved in the pre- and post-capacitation dependent detection of the various chemical cues.

## Materials and Methods

### Ethics Statement

#### Human

Human semen samples were obtained by masturbation from healthy volunteers with written informed consent and used in anonymous form. According to current German laws, no further approval was necessary for non-invasive recovery of samples from volunteers.

#### Animals

All experiments comply with *Principles of Animal Care*, publication no. 85-23, revised 1985, of the National Institutes of Health and with the current laws of Germany. Blood collection was approved by the regional government of Bavaria (Regierung Oberbayern), ID 55.2-1-54-2531.3-66-09. According to the *Protection of Animals Act* of Germany § 4 subpar. 3, killing of rodents and use of organs of sacrificed mice (“Toeten zu wissenschaftlichen Zwecken”) do not need any formal study approval. Due to this legislation, no *Animal Care and Use Committee* responsible for rodents exists at the institutions where the presented studies have been conducted, and ethic approval for animal use was neither necessary nor possible. Compliance to all German legislation and *Principles of Animal Care* was assured by a governmental assigned animal protection officer at the medical faculty at the University of Marburg or the University of Munich.

### Animals, general reagents and antisera

Male adult mice (129SV, C57BL/6and Balb/c) and rats (Wistar) were raised either in the animal facility of the medical faculty at the University of Marburg or the University of Munich. Animals were maintained at a 12 hour light/dark cycle with food and water ad libitum; mice were kept in individually ventilated cages (IVC) provided by Tecniplast (Hohenpeißenberg, Germany). Tas1r1-deficient Tas1r1-mCherry mice were kept on a mixed (129SV and C57BL/6) background (backcrossed to C57BL/6for up to 3 generations). Tas1r1-mCherry mice carry a recombinant *Tas1r1* allele, in which the Tas1r1 open reading frame is replaced by an mCherry expression cassette and will be described in detail elsewhere (Voigt et al., in preparation). Homozygous Tas1r1-mCherry mice are deficient of the Tas1r1 protein, but express Tas1r1 promoter-driven mCherry. Littermate wild-type animals or C57BL/6 mice were used as control animals, as indicated for each experiment. To visualize the mCherry protein, red fluorescence emission at 610 nm was detected after excitation with 590 nm.

To test for the specificity of taste receptor antisera generated against murine Tas1r isoforms, immunohistochemical analyses were performed with tissue sections of vallate papillae of the tongue. Antisera specific for the corresponding human taste receptors were analysed using HEK293 FlpIn T-REx cells (Invitrogen) heterologously expressing HSV-tagged taste receptor subtypes (for specification of plasmids see below “Western Blot Analyses”) in immunocytochemical and Western blot analyses. Additionally, if available, antigenic peptides used to generate taste receptor antibodies were applied to neutralize primary antisera. To this end, antisera were pre-treated with a 1–10 fold excess of the corresponding immunogenetic peptide; efficiency of neutralization was tested either in immunohistochemical analyses examining immunosignals on coronal sections of the tongue (murine antisera) or in Western blots and immunocytochemical studies using recombinant protein (human antisera).

The following antisera showed specific immunostaining: Rabbit polyclonal human-specific anti-Tas1r1 and anti-Tas1r3 antisera from Acris (Herford, Germany) (Tas1r1A), as well as rabbit anti-Tas1r3 antisera generated either against the mouse (Tas1r3M) (accession number NM_031872.2, amino acid 239–254) or the human (accession number NM_152228.1, amino acid 829–843) taste receptor subtype (Tas1r3hM) [53], kindly provided by R. Margolskee (Monell Chemical Senses Center, Philadelphia, USA); control immunogenic peptides for the latter antisera were synthesized by Thermo Electron (Ulm, Germany). Additionally, a mouse-specific rabbit anti-Tas1r3 IgG (Tas1r3A) (Abcam, Cambridge, UK) also showed specific labeling. Rabbit polyclonal antisera recommended for the detection of mouse, rat and human Tas1r1, Tas1r2 and Tas1r3 obtained from Santa Cruz Biotechnology (Heidelberg, Germany) as well as anti-Tas1r1 (*TR11*) and anti-Tas1r2 (*TR21*) antisera from Biotrend (Köln, Germany) showed no staining of sensory taste cells in our hands. Similar unspecific signals were obtained with a human-specific rabbit anti-Tas1r1 antiserum from Abnova (Taipei, Taiwan) and antisera against the human Tas1r1, Tas1r2 and Tas1r3 proteins from Genetex (Irvine, USA).

For immunodetection of the mCherry protein, a rabbit anti-dsred (red fluorescent protein from *Discosoma*) polyclonal antiserum from Clontech (California, USA) was used; the HSV epitope (Herpes simplex virus glycoprotein D) of heterologously expressed human taste receptors (s. below) was visualized using an anti-HSV antiserum from Novagen (Wisconsin, USA). Secondary antisera were the following: A fluorescein isothiocyanate (FITC)-conjugated goat anti-rabbit IgG was purchased from Sigma-Aldrich (Deisenhofen, Germany), the horseradish peroxidase (HRP)-conjugated goat anti-rabbit IgG was from Bio-Rad (Munich, Germany), HRP-conjugated sheep anti-mouse IgG was provided by GE Healthcare (Buckinhamshire, UK).

The nuclear staining dye TO-PRO-3 was from Invitrogen (Karlsruhe, Germany); tetramethyl-rhodamine-isothiocyanate (TRITC)-conjugated peanut agglutinin (TRITC-PNA), propidium iodide, the calcium ionophor A23187, percoll, Triton-100, Fura2-AM, laminin, poly-L-ornithine, laminin, aprotinin, DNAse, the phosphodiesterase inhibitor 3-isobutyl-1-methylxanthine (IBMX) [89], rolipram [124], zaprinast [89] as well as the taste stimuli L-glutamate sodium salt (MSG), inosine 5′-monophosphate disodium salt (IMP), saccharin and acesulfam K were purchased from Sigma Aldrich (Deisenhofen, Germany). The sweet-tasting plant protein thaumatin was kindly provided by E. Tareilus (Unilever, Rotterdam, Netherlands). Pluronic was obtained from Molecular Probes (Invitrogen, Karlsruhe, Germany), triethanolamine from Fluka (Sigma Aldrich, Deisenhofen, Germany), NP-40 alternative was from Calbiochem (Bad-Soden, Germany), sodium desoxycholate and tri-octylamine were purchased from Merck (Darmstadt, Germany). Primers were ordered from Metabion (Planegg-Martinsried, Germany); the non-radioactive enzyme immunoassay kit for cAMP determination (RPN 2251) was obtained from GE Healthcare. Unless specified otherwise, standard laboratory reagents were either purchased from Sigma-Aldrich (Deisenhofen, Germany) or Carl Roth (Karlsruhe, Germany).

### RNA isolation, cDNA synthesis and reverse transcription-polymerase chain reaction (RT-PCR)

Total RNA isolation from adult mouse testis and vallate papillae, excised from tongue epithelium was performed using the Nucleospin extraction method according to the manufacturer's protocol (Machery-Nagel, Düren, Germany). Isolated total RNA was subsequently reverse-transcribed using the Supercript III reverse transcriptase (Invitrogen GmbH, Karlsruhe, Germany) and oligo-dT primers according to the manufacturer's recommendations.

For the amplification of Tas1 taste receptor fragments by RT-PCR, reverse-transcribed cDNA from vallate papillae or testis tissue was initially examined for contamination with genomic DNA using exon spanning primer pairs of beta-actin [125] and the ribosomal gene L8 [126]. Amplification of the Tas1 receptor sequence was performed in a volume of 25 µl containing 2.5 µl 10×PCR buffer), 0.8 mM dNTPs (deoxyribonucleotide triphosphates), 0.125 µl Taq DNA polymerase (both Fermentas, St. Leon-Rot, Germany), 4 pmol of each primer and 1 µl of quality-checked cDNA. Optimized PCR conditions consisted of 35 cycles at 94°C for 1 min followed by annealing for 1 min followed by elongation at 72°C for 1 min. The PCR program finished with a final annealing period at 72°C for 7 min. Following PCR reactions, aliquots of amplicons (10 µl) were analyzed by electrophoresis on agarose gels, sub-cloned into pGEM-T easy vector (Promega, Mannheim, Germany) and sequenced to verify their identity (MWG Biotech, Ebersberg, Germany).

The following oligonucleotides were used:

Tas1r1 (GenBank accession no. AY032623, expected product size: 468 base-pairs [bp], TM (melting temperature) 61°C)

5′ ACGGCCATGGCTATCACCTCTTCC 3′ (forward) and

5′ CGCCCAGCTGCCCGTAGTCA 3′ (reverse)

Tas1r2 (GenBank accession no. AY032622, expected product size: 403 bp, TM 61°C)

5′ CTTTCGGGGGAGCGTGTGGTCTAC 3′ (forward) and

5′ ACGGGTGGAGGCCTATGGGTTTTT 3′ (reverse)

Tas1r2 alternative primer pair I (GenBank accession no. AY032622, expected product size: 851 bp, TM 60°C)

5′ CCTAACGAGACCAGCCTGAG3′ (forward) and

5′ CGGCAGAAACAGGAGAAGAC 3′ (reverse)

Tas1r2 alternative primer pair II (GenBank accession no. AY032622, expected product size: 581 bp, TM 60°C)

5′ CCCCCAACAACACGGTCCCCA 3′ (forward) and

5′ GGGCCCGTGGTAACGCATCC 3′ (reverse)

Tas1r3 (GenBank accession no. NM0311872, expected product size: 510 bp TM 60°C)

5′ TGAGCTGGGCAAACTGGCTA 3′ (forward) and

5′ TCTTGGCATTCCTTCCCAGG 3′ (reverse)

L8 (GenBank accession no NM 012053, expected product size: 406 bp; genomic DNA: 631 bp, TM 60°C)

5′ CCTACGTGCTGTGGACTTCGC 3′ (forward) and

5′ TCTGTTGGCAGAGGAAATGACC 3′ (reverse)

beta-Actin (GenBank accession no NM007393, expected product size: 425 bp; TM 60°C)

5′ GGCTACAGCTTCACCACCAC 3′ (forward) and

5′ GAGTACTTGGCGTCAGGAGG 3′ (reverse)

### Mouse tail DNA extraction and genotyping

Mouse tail biopsies were obtained by cutting the tip of the tail (0.5–1 mm) with small scissors at the time of weaning. Genomic DNA was isolated by treating the tissue with 1 mg/ml proteinase K (Roche, Basel, Switzerland) in 330 µl digestion buffer (25 mM EDTA, 75 mM NaCl, 1%% SDS, pH 8.0) at 55°C overnight. Subsequently, 15 µg RNAse A (Sigma-Aldrich, Deisenhofen, Germany) were added and the solution was incubated at 37°C for 30 min. After addition of 100 µl saturated NaCl solution (35%), probes were mixed at RT for 15 min, centrifuged for 30 min at 13,000 g, and the supernatant was transferred to a new reaction tube. 860 µl ethanol were added to the supernatant, mixed, and DNA was precipitated by centrifugation at 13,000 g for 15 min. The supernatant was discarded, and the pellet washed with 70% ethanol. After a final centrifugation at 3,000 g for 5 min, the washing solution was discarded, and the pellet air dried at RT, resuspended in H_2_O and stored at 4°C.

For PCR genotyping, DreamTaq DNA polymerase system from Fermentas (St. Leon-Rot, Germany) was used. 2.5 µl 10×PCR-buffer, 0.8 mMdNTPs, 0.125 µl Taq DNA polymerase, 4 pmol of each primer and 1 µl of isolated genomic tail DNA were mixed in a total amplification volume of 25 µl. PCR conditions consisted of 5 cycles at 94°C for 1 min, annealing at 65°C for 1 min and elongation at 72°C for 1 min. This amplification was followed by a second amplification cycle of 5 times 94°C for 1 min, annealing at 63°C for 1 min and elongation at 72°C for 1 min. A third amplification cycle was repeated 35 times and consisted of 94°C for 1 min, 61°C for 1 min and 72°C for 1 min. The PCR program finished with a final elongation period at 72°C for 7 min. Amplification products were separated on 1% agarose gels containing ethidium bromide and photographed for documentation.

Genotyping primers for the Tas1r1 mCherry mouse line:

wild-type Tas1r1(expected product size: 711 bp)

5′ GAATCCACCTGGTTTCCATCCACGTC 3′ (forward) and

5′ CTCTCAGGGTGACTTCAGTCTTTAGAGATGG 3′ (reverse)

mCherry knock-in (expected product size: 462 bp)

5′ GAATCCACCTGGTTTCCATCCACGTC 3′ (forward) and

5′ GTTGTTGGGGCACTCCATGTTGCT 3′ (reverse)

### Sperm preparation

Sperm from adult mice and rats were isolated as described previously [127]. Carefully dissected caudae epididymes were briefly washed in HS buffer (30 mM HEPES, 135 mM NaCl, 5 mM KCl, 2 mM CaCl_2_, 1 mM MgSO_4_, 10 mM glucose, 10 mM lactic acid, and 1 mM pyruvic acid, pH 7.4). Subsequently, tissue was transferred to HS supplemented with 0.5% BSA and 15 mM NaHCO_3_ (HS/BSA/NaHCO_3_) and incised several times to allow the sperm to exude into the medium. After a “swim out” period of 15 min at 37°C and 5% CO_2_, the medium was collected, sperm were concentrated by centrifugation (5 min, 400 g, RT), washed with PBS (150 mM NaCl, 1.4 mM KH_2_PO_4_, 8 mM Na_2_HPO_4_, pH 7.4) and used for immunofluorescence or Western blot analyses.

Freshly ejaculated human semen samples were obtained from young healthy donors. After liquefication at room temperature (RT) for 30 min, the ejaculate was covered with pre-warmed HS working solution (37°C), and sperm were allowed to swim up for 30 minutes. Subsequently, motile sperm in the supernatant were washed with PBS (5 min, 400 g, RT) and used for immunocytochemistry. For Western blot analyses, semen was washed twice with a nine fold volume of PBS. Sperm were then collected by centrifugation (5 min, 500 g, RT), quickly frozen in liquid nitrogen and stored at −80°C.

### Immunocytochemistry and Confocal Microscopy

For immuncytochemical analyses, sperm from different species were isolated as described above, placed on glass slides and allowed to settle for 15 min at RT. Adherent cells were washed with PBS and subsequently fixed with methanol or acetone/methanol. For methanol fixation, slides were put in ice-cold (−20°C) methanol for 2 min and then transferred directly to PBS [38]. For acetone/methanol fixation, cells were incubated for 10 min in acetone/methanol (1∶1, −20°C), air dried for 20 min at RT and washed with PBS.

The following steps were performed in a humidified chamber: To prevent non-specific binding of antibodies, samples were incubated for 30 min at RT with PBS supplemented with 10% fetal calf serum (FCS) (PAA laboratories, Pasching, Austria) and incubated thereafter with the primary antisera diluted in 10% FCS/PBS at 4°C overnight. To control antiserum specificity, primary antisera were pre-incubated with a 5 fold excess of the corresponding peptides used to generate the primary antibody for 30 min at 4°C; this pre-incubation was performed in PBS only and 10% FCS were added just prior to slide incubation. To check unspecific binding of the secondary antibody, control slides were incubated with 10% FCS/PBS only. After removing the primary antibodies by three washes with PBS (5 min), cells were incubated with a 1∶750 dilution of a FITC-conjugated secondary antibody for 1 h at RT. Subsequently, slides were washed three times with PBS, and cell nuclei were counterstained with propidium iodide [128] or TO-PRO-3 [129].

For acrosomal co-staining [41], cells were incubated with TRITC-coupled peanut agglutinin (TRITC-PNA, Sigma-Aldrich, Deisenhofen, Germany) diluted in 10% FCS/PBS for 30 min at RT after secondary antibody incubation. After removing excess of fluorescence-conjugated lectin by three washes with PBS, samples were coated with fluorescent mounting medium (DakoCytomation, Hamburg, Germany) and examined with a Zeiss LSM 510 Meta laser scanning confocal microscope and the Zeiss LSM image browser software (Carl Zeiss, Jena, Germany).

### Immunostaining of tissue sections

Immunohistochemical experiments were performed as outlined previously [106]. Briefly, freshly dissected vallate and fungiforme papillae, testes or epididymis from adult wild-type or mutant male mice were fixed in 4% paraformaldehyde diluted in 100 mM sodium-phosphate buffer (Na_2_HPO/NaH_2_PO_4_, pH 7.4) for 2 h at 4°C, cryo-protected at 4°C in 25% sucrose/PBS overnight and subsequently embedded in tissue freezing medium (Leica, Nussloch, Germany), frozen on dry ice and stored at −70°C. Coronal sections (6–10 µm) were cut at −18°C (CM 3050 S cryostat Leica, Microsystems, Wetzlar, Germany) and adhered to Superfrost plus microslides (Menzel Glaeser, Braunschweig, Germany).

Sections were air dried, washed in PBS and blocked for 30 min at RT with 10% normal goat serum and 0.3% Triton X-100 in PBS. Primary antisera, diluted in blocking solution, were incubated at 4°C overnight in a humid chamber. To test antiserum specificity, IgGs were pre-incubated with their immunogenic peptide (5 fold excess) for 30 min at RT before applying the neutralized primary antisera on the histological tissue sections. After three washes with PBS, slides were incubated with a 1∶750 dilution of the FITC-conjugated anti-rabbit IgG for 2 h at RT; control slides were only incubated with the diluted secondary antibody. Optional nuclear staining was performed adding TO-PRO-3 (Invitrogen, Darmstadt, Germany) in a dilution of 1∶1000 to the secondary antibody solution. After three washes with PBS slides were coated with fluorescent mounting medium and examined microscopically (see above).

### Western Blot Analyses

To test the specificity of antisera generated against the human Tas1rs, heterologously expressed human Tas1r proteins were used. To this aim, plasmids containing human taste receptor sequences as described in [111], [130], [131], [132] were utilized. Briefly, human Tas1r1 and Tas1r3 C-terminally fused to a herpes simplex virus (HSV) glycoprotein D epitope tag [130] and cloned into a pcDNA3/FRT/TO vector (Invitrogen, Karlsruhe, Germany) were stably transfected into HEK293 FlpIn T-REx cells (Invitrogen). Cell lines were cultured at 37°C with 5% CO_2_ in DMEM supplemented with 1% penicillin/streptomycin and 10% FCS (PAA laboratories, Pasching, Austria), and expression of the Tas1r-HSV constructs was induced using 0.5 µg/ml tetracycline (Sigma-Aldrich, Deisenhofen, Germany). For Western blot analyses, cells were seeded in 6-well plates and Tas1r expression was induced for 24 h. Thereafter, cells were washed with PBS, lysed in sample buffer and applied to SDS-polyacrylamide gelelectrophoresis (SDS PAGE). Separated proteins were subsequently electro-blotted onto nitrocellulose (GE Healthcare, Munich, Germany) using a semidry blotting system, and protein bands were stained with Ponceau S. Nitrocellulose sheets were then washed with TBST (10 mM Tris/HCl, pH 8.0, 150 mM NaCl and 0.05% Tween 20), and non-specific binding sites were blocked with 5% non-fat milk powder (Roth, Karlsruhe, Germany) in TBST. Subsequently, blots were incubated overnight at 4°C with primary antisera diluted in 3% milk powder in TBST. The next morning, unbound antiserum was removed by three washes with TBST and subsequently, nitrocellulose sheets were incubated for 1 h at RT with a horseradish-peroxidase-conjugated goat anti-rabbit IgG (1∶7,500 dilution in TBST with 3% milk powder). Following three washes with TBST, the ECL-system (Amersham Biosciences Freiburg, Germany) or the SuperSignal West Pico Chemiluminescent Substrate (Pierce/Thermo Scientific, Rockford, USA) were used to visualize bound antibodies and detected by Kodak X-OMat UV films (Kodak, Stuttgart, Germany) or an automated chemiluminescence system (Peqlab, Erlangen, Germany).

### Analysis of reproductive success

Mice were genotyped (see above) and bred in a monogamous mating system. Breeding pairs of the Tas1r1 mutant mouse lines were analyzed for average litter size and for the time needed to litter (first and subsequent litters) and compared to wild-type littermates. Pubs were genotyped as described above. Statistical analysis was done by comparing obtained genotype distributions to the expected Mendelian distributions using the chi square test. Results were considered statistically significant when p<0.05.

### Morphometric analysis of reproductive organs and spermatozoa

To assess gonad weight, mice were sacrificed by cervical dislocation, weighted and then reproductive tissues were dissected. Testes were weighted immediately after dissection as well as after complete drying in a 37°C incubator for 48 h (dry weight). Relative testis weight was determined as quotient of testicular weight and body weight.

To determine the number of mature sperm in the epidydymis, the caudal parts of the epididymis were transferred to 2 ml HS buffer (37°C), and sperm were completely extruded with a forceps under optical control. After a swim out time of 5 min at 37°C, sperm were collected, diluted 1∶40 (v/v) in water and counted in a Neubauer chamber (Brand, Wertheim, Germany).

The morphology and motility of mutant sperm were first analyzed routinely using a light microscope (Olympus CX 31). For a more quantitative analysis of sperm morphology, micrographs of fixed and Coomassie Brilliant blue G stained sperm (see below) were taken with a CX41 light microscope (Olympus, Hamburg, Germany). Subsequently, length, circumference and the area of the sperm head [133] were assessed using the “C*ell A*” software from Olympus (Hamburg, Germany) and analyzed statistically for each animal group (8–15 sperm from 5 different animals of each genotype). Data presented are mean values ± SEM.

For morphological analyses of reproductive organs and evaluation of apoptosis during spermatogenesis, mouse testes were fixed in Bouin's solution (Sigma-Aldrich, Deisenhofen, Germany) for 24 h at RT, washed in 70% Ethanol, dehydrated in an ascending ethanol series (70%, 80%, 96%, 2×100%) followed by Xylol (2×100%) and finally embedded in paraffin. Sections of 3 µm were cut on a microtome (Leica Microsystems, Wetzlar, Germany), re-hydrated in Xylol (Carl Roth, Karlsruhe Germany) and descending ethanol concentrations (2×100%, 80% and 70%) and washed twice with water.

To analyze testis morphology of mutant and wild-type testis, a Hematoxilin-Eosin (HE) staining was performed using routine procedure [134]. Briefly, re-hydrated tissue sections were incubated in Mayer's hemalaun solution (Carl Roth, Karlsruhe, Germany) at RT for 12 minutes, washed for 10 min in water, stained with an eosin y solution (0.5% aqueous solution, Carl Roth, Karlsruhe, Germany) for another 10 min and rinsed in water. Subsequently, sections were dehydrated using the above mentioned procedure and mounted with Eukitt (Sigma-Aldrich, Deisenhofen, Germany). Tissue staining was documented using a CX41 microscope from Olympus (Hamburg, Germany).

### TUNEL Assay

To compare the apoptosis rate in testis of wild-type and mutant mice, DNA fragmentation was measured using the TUNEL method (TdT-mediated dUTP-biotin nick end labeling) [135]. TUNEL staining was performed on paraffin-embedded histological tissue sections using an apoptosis detection kit from Roche (Basel, Switzerland) [62] according to the manufacturer's protocol. Briefly, after rehydration (see above), sections were treated with 20 µg/ml proteinase K in 10 mM Tris, pH 7.4 (Roche, Basel, Switzerland) for 30 min at 37°C and then washed two times with PBS. Subsequently, 50 µl of labeling mixture were applied to the slides, covered with parafilm (Pechiney Plastic Packaging, Chicago, USA) and incubated for 1 h at 37°C. After three washes with PBS, nuclei were counterstained with 0.1 µg/µl DAPI (4′,6-Diamidin-2′-phenylindol-dihydrochlorid, Sigma-Aldrich, Deisenhofen, Germany) for 30 min at RT to visualize seminiferous tubules, washed twice with PBS, coated with fluorescent mounting medium (DakoCytomation, Hamburg, Germany) and examined under a fluorescent microscope (Zeiss, Jena, Germany, Meta and Leica Microsystems, Wetzlar, Germany). TUNEL-positive cells were counted per microscopic visual field, and the number of seminiferous tubules was determined for each field as well. Shown data represent mean values ± SDM.

### Determination of serum testosterone levels

After cervical dislocation, animals were decapitated and blood was immediately collected in a petri dish and transferred to a reaction tube. Clotting was allowed for 20 min followed by a centrifugation step at 4500 g for 10 min at RT. Subsequently, serum was transferred to a new reaction tube and stored at −20°C. After thawing serum samples on ice, testosterone was extracted three times with the fivefold volume of diethyl ether. Solvent of the pooled organic phases was subsequently evaporated in a speed-vac centrifuge (Bachofer, Reutlingen, Germany). The amount of extracted testosterone was then determined using the testosterone EIA kit from Cayman Chemicals (Ann Arbor, USA) according to the recommendations of the manufacturer. Absorbance of the used Ellman's reagent was measured using a plate reader (Fluostar Omega, BMG Labtech, Offenburg, Germany) and testosterone concentrations were calculated comparing absorption of serum probes with a testosterone standard curve.

### Determination of capacitation by cholesterol depletion

To determine the efficiency of capacitation, isolated spermatozoa were diluted in HS buffer supplemented with 0.5% BSA and 15 mM NaHCO_3_ (capacitation buffer, HS/BSA/NaHCO_3_), distributed to single reaction tubes and incubated for different time periods at 37°C in an atmosphere of 5% CO_2_. Every 30 min, one of the tubes was taken and immediately centrifuged at 400 g for 5 min. The supernatant was transferred to a new reaction tube and kept frozen (−20°C) until cholesterol determination. Cholesterol concentrations in the collected supernatants were measured as described recently [34] using the *Amplex red system* from Invitrogen (Karlsruhe, Germany), according to the manufacturer's recommendations. Briefly, 50 µl of supernatant were mixed with 50 µl Amplex red solution, incubated for 15 min at 37°C and measured in a Fluostar Omega (BMG Labtech, Offenburg, Germany) with excitation/emission wavelengths of 530/590 nm. The amount of cholesterol in each sample was determined using a cholesterol standard curve and presented as cholesterol depletion per cell.

### Sperm motility analysis

Casa motility assays were performed as described recently [136]. Briefly, after cervical dislocation, cauda epididymes were isolated, washed once in physiological NaCl solution, (0.9% in H_2_O) freed of fat and connecting tissue and then transferred to 500 µl HTF (artificial human tubular fluid). After cutting the epididymis, sperm were allowed to swim out for 5 min at 37°C. Subsequently, 10 µl of this sperm suspension were transferred to a 500 µl drop of HTF covered with mineral oil and incubated at 37°C for 10 min. Computer-assisted sperm analysis (CASA) was then performed using an IVOS sperm analyzer (Hamilton Thorne Research, Beverly, USA). Following parameters were recorded (units): motility (% of total), progressive sperm (% of total), average path velocity (VAP) (µm/sec), straight line velocity (VSL) (µm/sec), curvilinear velocity (µm/sec), amplitude of lateral head displacement (µm), beat cross frequency (hertz), straightness (quotient of VSL and VAP) (%) and linearity (quotient of VSL and VCL) (%). For each measurement, 30 frames were analyzed in 0.5 sec; 6 measurements with a total of at least 2000 spermatozoa were performed for each animal. Data shown represent mean values ± SEM of three littermate animals for each genotype, analyzed in independent experiments.

### Acrosome reaction assays

To study acrosome reaction, mouse sperm were isolated as described above and incubated in HS/BSA/NaHCO_3_ at 37°C/CO_2_ for 90 minutes to ensure full capacitation. As positive control for acrosome reaction, sperm were stimulated with 10 µM of the calcium ionophor A23187 dissolved in DMSO for 30 min at 37°C as described previously [128]. As negative control, aliquots of cells were incubated in parallel with dilutions of DMSO alone. To determine spontaneous acrosome reaction rates, sperm samples diluted in capacitation medium were fixed directly after isolation of spermatozoa (uncapacitated) or after 90 min incubation (capacitated). To analyze if tastants are able to imitate the z*ona pellucida* and induce acrosome reaction, 100–200 µl of capacitated sperm were incubated with different taste stimuli (MSG, IMP, NaCl, glucose, saccharin, acesulfame K, saccharin or thaumatin) for 30 min at 37°C. After incubation with the stimulus, cells were fixed with cell fixative (20 mM Na_2_HPO_4_, 150 mM NaCl, 7.5% formaldehyde) for at least 30 min, washed with post fix buffer (100 mM ammonium acetate, pH 9.0) and air-dried on glass slides. Induction of acrosome reaction was assessed by monitoring the intactness of the acrosome by Coomassie Blue G staining [137]. Acrosomal status was determined for at least 200 cells using an Olympus CX41 microscope equipped with bright-field light optics.

### Induction of acrosome reaction with z*ona pellucida*

ZP glycoproteins were prepared as described recently [138]. Briefly, mice ovaries were homogenized in HB complete medium (150 mM NaCl, 1 mM MgCl_2_, 1 mM CaCl_2_, 25 mM triethanolamin, 0.2 mg/ml aprotinin, 0.2 mg/ml DNase, 1% Nonidet NP-40 alternative, 1% sodium deoxycholate and complete protease inhibitor cocktail [Roche, Basel, Switzerland], pH 8.5), and the homogenate was fractionated on a 3-step percoll gradient (2%/10%/22% in HB complete medium). After centrifugation at 200 g and 4°C for 2 h, the “10%-percoll fraction” containing z*ona pellucida* (ZP) was collected and diluted with HB complete medium. ZPs were concentrated by subsequent centrifugation steps (16,000 g, 4°C). An aliquot of the pooled ZP suspension was applied on a glass slide and isolated ZPs were counted using a light microscope. For extraction of soluble ZP glycoproteins, ZPs were washed with 2.5 mM sodium phosphate (pH 7.0) buffer, resuspended in 5 mM sodium phosphate (pH 2.5) and heated to 60°C for 20 min. Thereafter, insoluble material and non-solubilized ZP were removed by centrifugation (16,000 g for 1 min), and the supernatant was collected. Heat solubilization was repeated once and the pooled supernatants were diluted with 2×HS/BSA/NaHCO_3_.

To induce acrosome reaction, sperm were capacitated for 90 min (see above), and subsequently incubated with 10 ZP/µl for 30 min at 37°C. As a negative control, samples were incubated with the sodium phosphate buffer used for heat solubilization of zonae (see above). After z*ona pellucida* stimulation, sperm were fixed, washed, air-dried on glass slides and assessed for acrosomal status as described above.

### Single Cell Calcium Imaging

To determine intracellular calcium concentrations in the sperm head, mouse spermatozoa were isolated as described above and either directly used for calcium imaging or capacitated for 60 min in capacitation buffer (HS/BSA/NaHCO_3_) prior to imaging. Subsequently, cells were centrifuged at 400 g for 5 min at RT and washed twice with HS (uncapacitated) or HS/NaHCO_3_ (capacitated) respectively. Cells were resuspended in 1 ml HS or HS/NaHCO_3_ containing Pluronic (0.003% final concentration) and 25 µM Fura-2AM and then incubated for 30 min at RT. After two washes with HS or HS/NaHCO_3_ and centrifugation at 400 g for 5 min, cells were resuspended in HS or HS/NaHCO_3_ (uncapacitated and capacitated, respectively) and incubated for 30 min at RT to allow de-esterification of the dye before starting the measurement. Sperm cells were then adhered to coverslips coated with laminin (50 µg/ml in PBS) and poly-L-ornithin (0.01% in H_2_O) for 2 min, and subsequently washed twice with HS/NaHCO_3_ to eliminate non-adherent cells. Fluorimetric determination of calcium concentrations in the sperm head was performed using a Polychrome V monochromator (Till-Photonics, Gräfelfing, Germany) and an Andor charge-coupled device camera coupled to an inverted microscope (IX71, Olympus, Hamburg, Germany). Fura-2 ratios were determined as quotient of the detected fluorescence intensities at 510 nm after excitation with 340 nm or 380 nm, respectively (TillVisionSoftware, Till-Photonics, Gräfelfing, Germany). Only cells showing a significant increase in Fura-2 ratio in response to 5 µM ionomycinapplicated at the end of each measurement were included in the calculations. To convert Fura-2 ratios to intracellular calcium concentrations, cells were treated with 10 mM EGTA in HS buffer (without calcium) with 5 µM Ionomycin, followed by stimulation with 60 mM CaCl_2_ to determine minimal and maximal fluorescence ratios, respectively. Calcium concentrations were then calculated according to Grynkiewicz [84].

### Determination of intracelluar Ca^2+^ concentrations in sperm populations upon glutamate stimulation

To determine cytoplasmatic Ca^2+^ concentrations, mouse sperm were isolated, capacitated in HS/BSA/NaHCO_3_ for 60 min and loaded with Fura-2AM as described above. After a recovery period of 30 min at RT, sperm suspension was distributed on a 96-well plate (90 µl per well, containing 4–10×10^6^ cells/ml) and total fluorescence emission (520±20 nm) was measured after excitation of the sample with 340±15 nm or 380±15 nm in a Fluostar Omega plate reader (BMG Labtech, Offenburg, Germany). Detector gain sensitivity was adjusted to yield a basal Fura-2 ratio (F340/F380) of 1. To stimulate cells, 10 µl of each test substance (MSG and ionomycin, dissolved in HS/NaHCO_3_), were automatically injected 10 sec after starting the measurement into 90 µl of buffer containing sperm. Stimulation with buffer alone was used to exclude effects of the injection itself.

### Measurement of cAMP Concentration in Sperm

Intracellular cAMP concentrations were determined as described previously [139] with double samples for each condition. Briefly, freshly isolated spermatozoa were allowed to swim out of the cut epididymis, either for 15 min in HS buffer (uncapacitated) or for 60 min in HS buffer supplemented with BSA and NaHCO_3_ (capacitated). Uncapacitated sperm were subsequently washed with HS before the reaction was started by mixing 100 µl of pre-warmed HS-buffer or HS supplemented with the relevant test substances (10 mM MSG, 0.5 µM IBMX, 50 mM NaHCO_3_) with 100 µl of spermatozoa (ca. 1×10^6^) and incubated for 5 min at37°C. Capacitated sperm were treated analogously using HS/NaHCO_3_ for washing and dissolving of test substances. After stopping the reaction by shock freezing in liquid nitrogen, 100 µl of ice-cold perchloric acid (7%) was added and quenched samples were neutralized as described previously [139]. cAMP concentrations were determined using a non-radioactive cAMP kit (RPN2251, GE Healthcare, Munich, Germany), based on the competition between unlabeled cAMP in the sample and a fixed quantity of peroxidase-labeled cAMP [140]. The indicated concentrations of the different modulators in the results section represent concentrations during incubation of sperm. DMSO used to dilute IBMX never exceeded 0.5% [v/v]; sperm preparations which did not show at least 1.5 fold cAMP accumulation in wild type animals upon IBMX stimulation were excluded from analysis. Optical density of each individual sample was measured at 450 nm using a Fluostar Omega plate reader (BMG Labtech, Offenburg, Germany); mean values of measured extinctions were used to calculate cAMP concentration in the individual probes; subsequently amount of cAMP was corrected for the number of sperm in each sample.

### Statistical analyses

Unless stated otherwise, statistical analyses were performed using the Student's t-test. A p-value≤0.05 was considered to be statistically significant.

## Supporting Information

Figure S1 **Amplification of Tas1r2-transcripts in cDNA from murine vallate papillae and testicular tissue using RT-PCR.** An alternative primer pair matching the published sequence of mouse Tas1r2 was applied using cDNA derived from vallate papillae of the tongue ([*VP+RT*]) and testicular cDNA ([*Te+RT*]). Probes lacking the reverse transcription enzyme [−RT] and water were used as negative control. Note that an amplification product of the expected size (581 bp) was obtained from reverse transcribed taste cDNA only ([*VP+RT*]), whereas the testis cDNA and the non-transcribed probes did not show any PCR product. The corresponding 500-bp DNA marker is shown on the left.
(TIF)

Figure S2 **Specification of subtype-specific antisera for human Tas1r1 and Tas1r3.** [**A** and **B**] Identification of members of Tas1 taste receptor family by Western Blot analysis. Total cell preparations of HEK 293 cells heterologously expressing human Tas1r1 [**A**] or Tas1r3 [**B**] were separated by SDS-PAGE and subsequently probed with an anti-Tas1r1 antiserum or the anti- Tas1r3A-IgG (ab, left lanes). Application of the Tas1r1 specific antiserum to lysates of Tas1r1 expressing cells resulted in one single band of the expected size (93 kDa; [**A**], left lane; [*ab*]), which was prevented by pre-incubation of the antiserum with its neutralizing peptide ([**A**], right lane; [*ab+bp*]). A comparable result was seen for the Tas1r3A antiserum [**B**] which led to an immunoreactive band of about 110 kDa ([**B**], left lane; [*ab*]) after applying the antiserum. This immunoreactive band was also completely abolished by the immunogenic peptide ([**B**], right lane; [*ab+bp*]). The positions of the molecular weight standards [MW] in kDa are indicated on the right. [**C**] Immunocytochemical analysis of Tas1r3 expression in human sperm. Ejaculated human sperm were incubated with one of the two human specific Tas1r3 antisera (Tas1r3A and Tas1r3M); bound primary antiserum was visualized applying a FITC-conjugated anti-rabbit IgG. The representative confocal micrographs document that the anti-Tas1r3 IgG ([*Tas1r3 M*]) showed a staining in the flagellum (arrow) and in the acrosomal region (middle panels; [*Tas1r3 M*]) as well as at the equatorial segment (right panel in the middle; [*Tas1r3M*, arrowhead). The Tas1r1A antiserum shows a weaker staining which was mainly concentrated in the equatorial segment (upper panels; [*Tas1r3A*, arrowheads]). This labeling was completely eliminated upon neutralizing the primary antiserum with an excess of the corresponding immunogenic peptide (lower panels; [*Tas1r3A+BP*]). Negative controls, in which the primary antiserum was omitted, did not show any labeling (data not shown). Confocal images were produced by an overlay of corresponding fluorescence channels (propidium iodide, [red]; FITC-conjugated secondary antiserum, [green]) and the transmission channel. Boxes indicate regions that are magnified in insets in the right panels. Experiments were repeated with at least three independent sperm preparations from different donors, which showed comparable results.
(TIF)

Figure S3 **Effect of monosodium glutamate and sweet tastants on acrosome reaction.** [**A**] Acrosome reaction in sperm of Tas1r1 null mice is not affected by Monosodium-glutamate. To evaluate whether the tastant MSG and the allosteric modulator IMP influence acrosome reaction in spermatozoa and whether this signaling is lost upon Tas1r1 deletion, epididymal capacitated sperm of animals of wild-type and Tas1r1-deficient animals were incubated for 30 min with either MSG (10 mM), IMP (1 mM), a mixture of the two tastants or with 10 mM NaCl to assess the effect of increased sodium concentrations. Quantifying the acrosomal status of treated sperm revealed that neither MSG nor the combination of MSG and IMP elicited an elevation in the percentage of acrosome reaction in wild-type and Tas1r1 null sperm. Data calculated as percentages of acrosome reacted sperm represent mean values ± SEM of 7 independent experiments of different mouse sperm preparations of littermate animals and animals with identical strain background of both genotypes. [**B**] Effect of sweet compounds on acrosome reaction. To investigate whether sweet substances might induce acrosomal secretion in sperm cells, capacitated spermatozoa of wild-type animals were treated for 30 min with 100 mM glucose, 1 mM saccharin, 100 mM acesulfam K or 100 µM thaumatin; subsequently, acrosomal status was determined as described above. Comparing acrosome reaction rates of the tested sweet tastants, no significant difference (p≤0.05) was observed compared to the spontaneous acrosome reaction rate [*basal*]. Data shown represent mean values ± SEM of 3–7 independent experiments.
(TIF)

Figure S4 **Effect of monosodium glutamate on cAMP levels in wild-type and Tas1r1-deficient sperm.** Isolated epididymal sperm of wild-type [*+/+*] and Tas1r1-deficient [*−/−*] mice were either capacitated [*capacitated*] or left uncapacitated [*uncapacitated*] and treated with buffer alone ([*basal*], white columns) or with 10 mM MSG ([*MSG*], grey columns) for 5 min at 37°C. Subsequently, stimulation was stopped by shock-freezing the cells in liquid nitrogen and cAMP was extracted with PCA (7%), and quantified using a commercially available EIA kit. In uncapacitated wild-type sperm, MSG [*MSG*] induced a significant increase in cAMP concentration compared to basal cAMP levels ([*+/+*], left column pair). In Tas1r1 null sperm [−/−] basal cAMP is already elevated to the same extent registered in wild-type sperm and did not further increase upon addition of MSG. The MSG induced cAMP signal was only detected in uncapacitated wild-type spermatozoa; upon *in vitro* capacitation, sperm of the two genotypes did not show significant effects upon MSG application [*MSG*] compared to buffer alone (two right column pairs, [*basal*]). Data shown represent mean values ± SEM of 9–11 independent sperm preparations of each genotype.
(TIF)

Table S1 **Comparison of basal cAMP concentration in uncapacitated sperm of wild-type and Tas1r1-deficient mice.** Epididymal sperm of wild-type [*+/+*] and Tas1r1-deficient [*−/−*] littermates and cousins (identical genetic background, same age) were isolated in parallel, incubated for 20 min in HS buffer and subsequently assayed for their cAMP content. cAMP concentrations [fmol/10^6^ cells] determined for each animal pair are presented as means ± SEM in ascending order; statistical significance of the data (p values) was calculated employing a paired student's T-Test of corresponding mouse pairs (p = 0.023). In addition, data (right column) and statistical significance were calculated as % of cAMP determined for wild-type sperm (p = 0.015). Note that although absolute cAMP concentrations broadly vary between sperm of individual animals of one genotype, only two out of 15 pairs show lower cAMP levels in Tas1r1 deficient sperm when compared to the related wild-type cells.
(DOC)

Table S2 **Effects of different PDE inhibitors on cAMP accumulation in uncapacitated spermatozoa of wild-type and Tas1r1 null sperm.** Epididymal sperm of wild-type [*+/+*] and Tas1r1-deficient [*−/−*] mice were isolated in HS (for 15 min) and treated for 5 min at 37°C with buffer alone [*basal*], 0.5 mM IBMX [*IBMX*] or the PDE-4 selective inhibitor rolipram [*rolipram*, 10 µM] (n = 3–4). Although rolipram only slightly increases basal cAMP compared to IBMX, cAMP concentrations were adjusted in sperm of both genotypes upon application of the two PDE blockers.
(DOC)
