# Supplementary figures and images for: Expression of Tas1 Taste Receptors in Mammalian Spermatozoa: Functional Role of Tas1r1 in Regulating Basal Ca2+ and cAMP Concentrations in Spermatozoa
Source: PLoS One. 2012 Feb 29;7(2):e32354. doi: 10.1371/journal.pone.0032354 (PMC3303551; doi:10.1371/journal.pone.0032354)

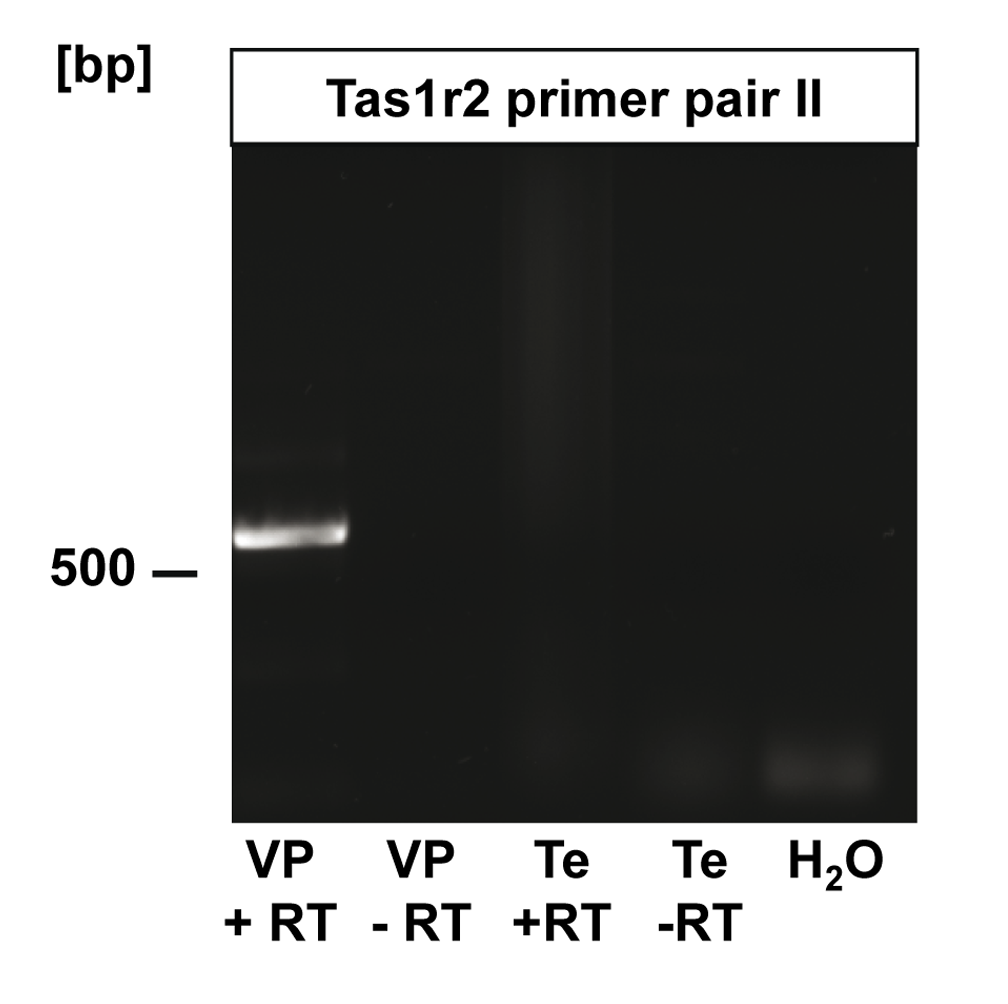

Supplement: Figure S1 — Amplification of Tas1r2-transcripts in cDNA from murine vallate papillae and testicular tissue using RT-PCR. An alternative primer pair matching the published sequence of mouse Tas1r2 was applied using cDNA derived from vallate papillae of the tongue ([VP+RT]) and testicular cDNA ([Te+RT]). Probes lacking the reverse transcription enzyme [−RT] and water were used as negative control. Note that an amplification product of the expected size (581 bp) was obtained from reverse transcribed taste cDNA only ([VP+RT]), whereas the testis cDNA and the non-transcribed probes did not show any PCR product. The corresponding 500-bp DNA marker is shown on the left. (TIF) [file pone.0032354.s001.tif]

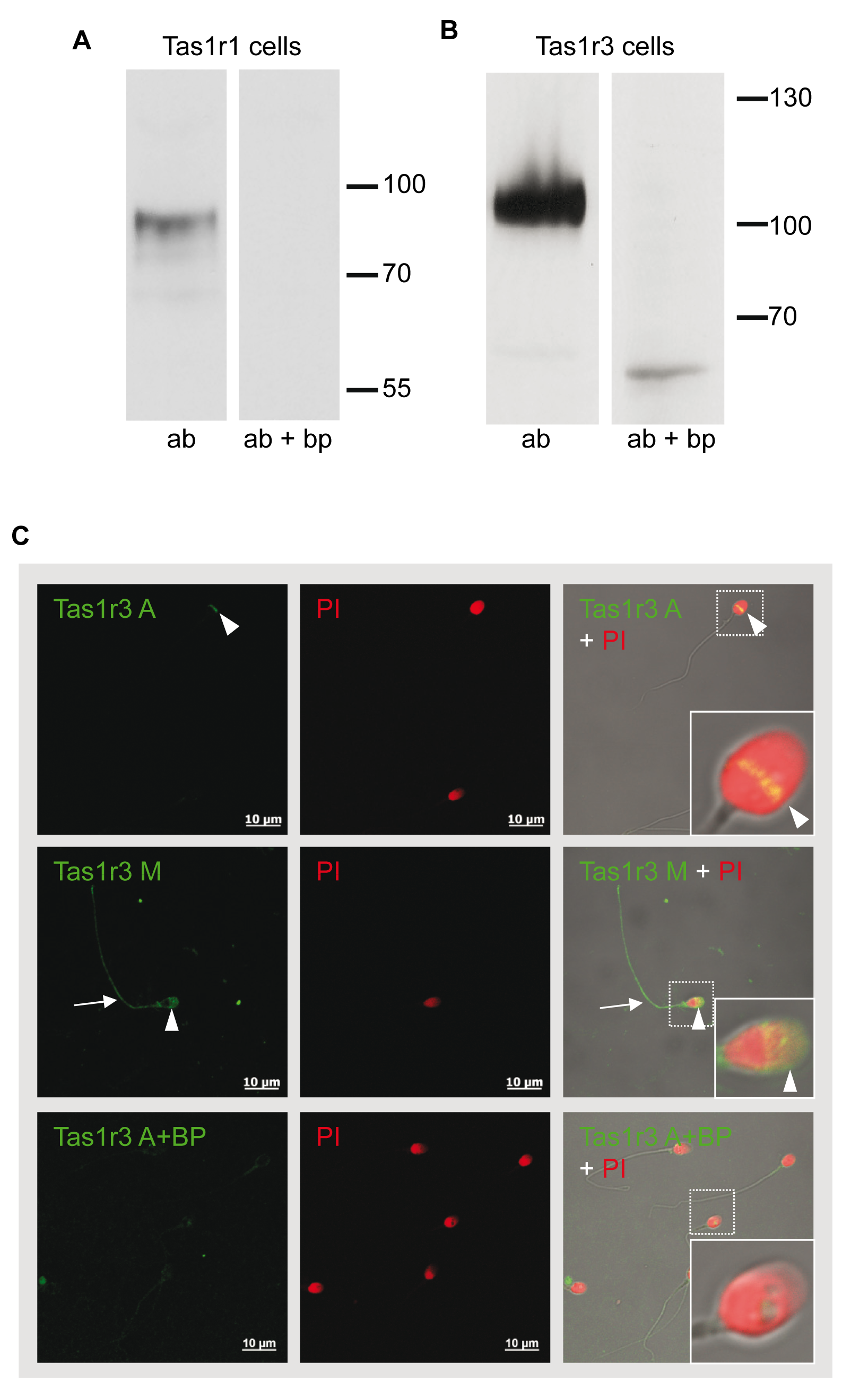

Supplement: Figure S2 — Specification of subtype-specific antisera for human Tas1r1 and Tas1r3. [A and B] Identification of members of Tas1 taste receptor family by Western Blot analysis. Total cell preparations of HEK 293 cells heterologously expressing human Tas1r1 [A] or Tas1r3 [B] were separated by SDS-PAGE and subsequently probed with an anti-Tas1r1 antiserum or the anti- Tas1r3A-IgG (ab, left lanes). Application of the Tas1r1 specific antiserum to lysates of Tas1r1 expressing cells resulted in one single band of the expected size (93 kDa; [A], left lane; [ab]), which was prevented by pre-incubation of the antiserum with its neutralizing peptide ([A], right lane; [ab+bp]). A comparable result was seen for the Tas1r3A antiserum [B] which led to an immunoreactive band of about 110 kDa ([B], left lane; [ab]) after applying the antiserum. This immunoreactive band was also completely abolished by the immunogenic peptide ([B], right lane; [ab+bp]). The positions of the molecular weight standards [MW] in kDa are indicated on the right. [C] Immunocytochemical analysis of Tas1r3 expression in human sperm. Ejaculated human sperm were incubated with one of the two human specific Tas1r3 antisera (Tas1r3A and Tas1r3M); bound primary antiserum was visualized applying a FITC-conjugated anti-rabbit IgG. The representative confocal micrographs document that the anti-Tas1r3 IgG ([Tas1r3 M]) showed a staining in the flagellum (arrow) and in the acrosomal region (middle panels; [Tas1r3 M]) as well as at the equatorial segment (right panel in the middle; [Tas1r3M, arrowhead). The Tas1r1A antiserum shows a weaker staining which was mainly concentrated in the equatorial segment (upper panels; [Tas1r3A, arrowheads]). This labeling was completely eliminated upon neutralizing the primary antiserum with an excess of the corresponding immunogenic peptide (lower panels; [Tas1r3A+BP]). Negative controls, in which the primary antiserum was omitted, did not show any labeling (data not shown). Confocal images were pr [file pone.0032354.s002.tif]

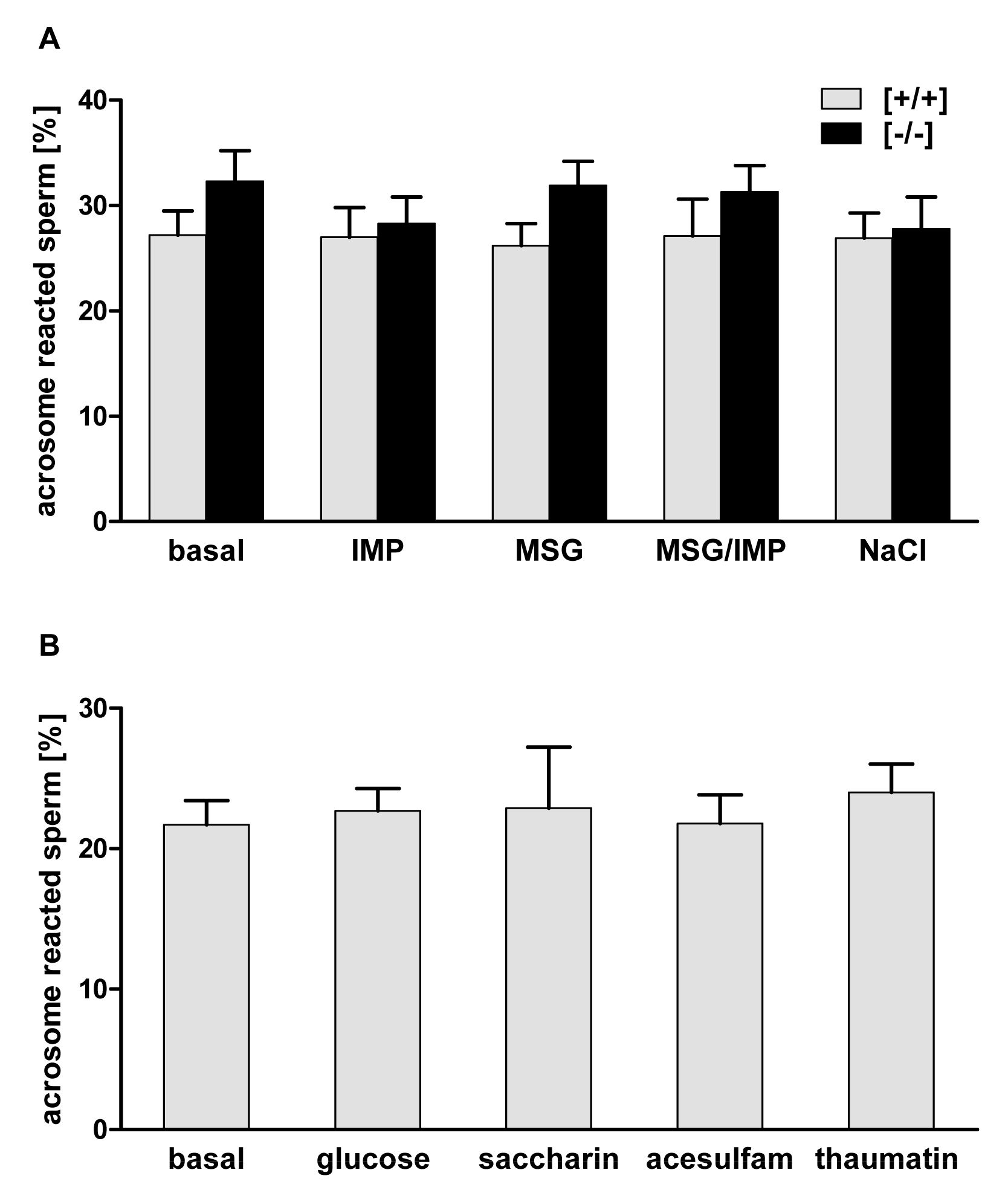

Supplement: Figure S3 — Effect of monosodium glutamate and sweet tastants on acrosome reaction. [A] Acrosome reaction in sperm of Tas1r1 null mice is not affected by Monosodium-glutamate. To evaluate whether the tastant MSG and the allosteric modulator IMP influence acrosome reaction in spermatozoa and whether this signaling is lost upon Tas1r1 deletion, epididymal capacitated sperm of animals of wild-type and Tas1r1-deficient animals were incubated for 30 min with either MSG (10 mM), IMP (1 mM), a mixture of the two tastants or with 10 mM NaCl to assess the effect of increased sodium concentrations. Quantifying the acrosomal status of treated sperm revealed that neither MSG nor the combination of MSG and IMP elicited an elevation in the percentage of acrosome reaction in wild-type and Tas1r1 null sperm. Data calculated as percentages of acrosome reacted sperm represent mean values ± SEM of 7 independent experiments of different mouse sperm preparations of littermate animals and animals with identical strain background of both genotypes. [B] Effect of sweet compounds on acrosome reaction. To investigate whether sweet substances might induce acrosomal secretion in sperm cells, capacitated spermatozoa of wild-type animals were treated for 30 min with 100 mM glucose, 1 mM saccharin, 100 mM acesulfam K or 100 µM thaumatin; subsequently, acrosomal status was determined as described above. Comparing acrosome reaction rates of the tested sweet tastants, no significant difference (p≤0.05) was observed compared to the spontaneous acrosome reaction rate [basal]. Data shown represent mean values ± SEM of 3–7 independent experiments. (TIF) [file pone.0032354.s003.tif]

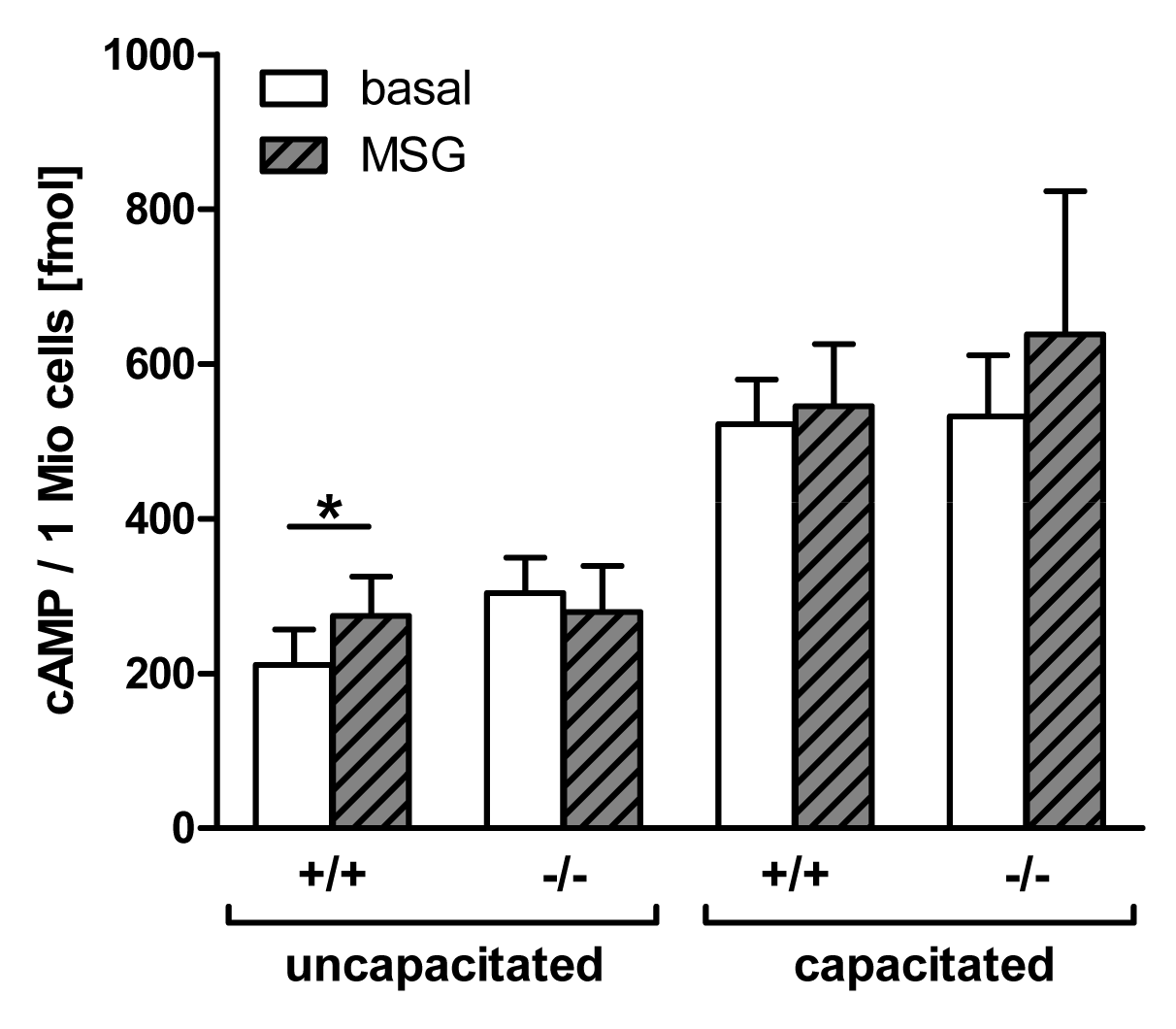

Supplement: Figure S4 — Effect of monosodium glutamate on cAMP levels in wild-type and Tas1r1-deficient sperm. Isolated epididymal sperm of wild-type [+/+] and Tas1r1-deficient [−/−] mice were either capacitated [capacitated] or left uncapacitated [uncapacitated] and treated with buffer alone ([basal], white columns) or with 10 mM MSG ([MSG], grey columns) for 5 min at 37°C. Subsequently, stimulation was stopped by shock-freezing the cells in liquid nitrogen and cAMP was extracted with PCA (7%), and quantified using a commercially available EIA kit. In uncapacitated wild-type sperm, MSG [MSG] induced a significant increase in cAMP concentration compared to basal cAMP levels ([+/+], left column pair). In Tas1r1 null sperm [−/−] basal cAMP is already elevated to the same extent registered in wild-type sperm and did not further increase upon addition of MSG. The MSG induced cAMP signal was only detected in uncapacitated wild-type spermatozoa; upon in vitro capacitation, sperm of the two genotypes did not show significant effects upon MSG application [MSG] compared to buffer alone (two right column pairs, [basal]). Data shown represent mean values ± SEM of 9–11 independent sperm preparations of each genotype. (TIF) [file pone.0032354.s004.tif]
