# Supplementary material for: Expression of Tas1 Taste Receptors in Mammalian Spermatozoa: Functional Role of Tas1r1 in Regulating Basal Ca2+ and cAMP Concentrations in Spermatozoa
Source: PLoS One. 2012 Feb 29;7(2):e32354. doi: 10.1371/journal.pone.0032354 (PMC3303551; doi:10.1371/journal.pone.0032354)
Supplement: Table S1 — Comparison of basal cAMP concentration in uncapacitated sperm of wild-type and Tas1r1-deficient mice. Epididymal sperm of wild-type [+/+] and Tas1r1-deficient [−/−] littermates and cousins (identical genetic background, same age) were isolated in parallel, incubated for 20 min in HS buffer and subsequently assayed for their cAMP content. cAMP concentrations [fmol/106 cells] determined for each animal pair are presented as means ± SEM in ascending order; statistical significance of the data (p values) was calculated employing a paired student's T-Test of corresponding mouse pairs (p = 0.023). In addition, data (right column) and statistical significance were calculated as % of cAMP determined for wild-type sperm (p = 0.015). Note that although absolute cAMP concentrations broadly vary between sperm of individual animals of one genotype, only two out of 15 pairs show lower cAMP levels in Tas1r1 deficient sperm when compared to the related wild-type cells. (DOC) [file pone.0032354.s005.doc]

**Table S1: Comparison of basal cAMP concentration in uncapacitated sperm of wild-type and Tas1r1-deficient mice.**

|  | **cAMP concentration [fmol/10^6^ cells]** | | **[%] of WT** |
| --- | --- | --- | --- |
| **animal pair** | **[+/+]** | **[-/-]** | **[-/-]** |
| 1 | 49 | 74 | 151 |
| 2 | 78 | 84 | 108 |
| 3 | 86 | 213 | 248 |
| 4 | 142 | 291 | 205 |
| 5 | 175 | 287 | 164 |
| 6 | 178 | 225 | 126 |
| 7 | 216 | 276 | 128 |
| 8 | 224 | 248 | 111 |
| 9 | 243 | 247 | 102 |
| 10 | 245 | 314 | 128 |
| 11 | 256 | 286 | 112 |
| 12 | 296 | 157 | 53 |
| 13 | 325 | 473 | 146 |
| 14 | 420 | 398 | 95 |
| 15 | 519 | 628 | 121 |
| **mean ± SEM** | **230 ± 33** | **280 ± 36** | **133 ± 12 %** |
| **p-value**  **paired T-test** | **0.023** | | **0.015** |
