# Supplementary material for: Expression of Tas1 Taste Receptors in Mammalian Spermatozoa: Functional Role of Tas1r1 in Regulating Basal Ca2+ and cAMP Concentrations in Spermatozoa
Source: PLoS One. 2012 Feb 29;7(2):e32354. doi: 10.1371/journal.pone.0032354 (PMC3303551; doi:10.1371/journal.pone.0032354)
Supplement: Table S2 — Effects of different PDE inhibitors on cAMP accumulation in uncapacitated spermatozoa of wild-type and Tas1r1 null sperm. Epididymal sperm of wild-type [+/+] and Tas1r1-deficient [−/−] mice were isolated in HS (for 15 min) and treated for 5 min at 37°C with buffer alone [basal], 0.5 mM IBMX [IBMX] or the PDE-4 selective inhibitor rolipram [rolipram, 10 µM] (n = 3–4). Although rolipram only slightly increases basal cAMP compared to IBMX, cAMP concentrations were adjusted in sperm of both genotypes upon application of the two PDE blockers. (DOC) [file pone.0032354.s006.doc]

**Table S2: Effects of different PDE inhibitors on cAMP accumulation in uncapacitated spermatozoa of wild-type and Tas1r1 null sperm.**

|  | **cAMP concentration [fmol/10^6^ cells]** | |
| --- | --- | --- |
| **conditions** | **[+/+]** | **[-/-]** |
| basal | 242 ± 7 | 274 ± 16 |
| IBMX (500 µM) | 457 ± 22 | 449 ± 50 |
| rolipram (10 µM) | 268 ± 5 | 260 ± 7 |
